# Multidrug Efflux Pumps from Enterobacteriaceae, *Vibrio cholerae* and *Staphylococcus aureus* Bacterial Food Pathogens

**DOI:** 10.3390/ijerph120201487

**Published:** 2015-01-28

**Authors:** Jody L. Andersen, Gui-Xin He, Prathusha Kakarla, Ranjana KC, Sanath Kumar, Wazir Singh Lakra, Mun Mun Mukherjee, Indrika Ranaweera, Ugina Shrestha, Thuy Tran, Manuel F. Varela

**Affiliations:** 1 Department of Biology, Eastern New Mexico University, Portales, NM 88130, USA; E-Mails: jodyandersen@rocketmail.com (J.L.A.); prathusha.kakarla@enmu.edu (P.K.); najranaa@gmail.com (R.K.C.); munmun.mukherjee@enmu.edu (M.M.M.); indrika.ranaweera@enmu.edu (I.R.); ugina.shrestha@enmu.edu (U.S.); 2 Department of Clinical Laboratory and Nutritional Sciences, University of Massachusetts Lowell, Lowell, MA 01854, USA; E-Mails: Guixin_He@uml.edu (G.H.); ThuyTrang_Tran@student.uml.edu (T.T.); 3 QC Laboratory, Harvest and Post-Harvest Technology Division, Central Institute of Fisheries Education (CIFE), Seven Bungalows, Versova, Andheri (W), Mumbai 400061, India; E-Mails: sanathkm@yahoo.com (S.K.); wslakra@cife.edu.in (W.S.L.)

**Keywords:** antimicrobial, bacteria, resistance, efflux pumps, multidrug resistance, food pathogens, *Staphylococcus aureus*, Enterobacteriaceae, Vibrionaceae, infection

## Abstract

Foodborne illnesses caused by bacterial microorganisms are common worldwide and constitute a serious public health concern. In particular, microorganisms belonging to the Enterobacteriaceae and Vibrionaceae families of Gram-negative bacteria, and to the *Staphylococcus* genus of Gram-positive bacteria are important causative agents of food poisoning and infection in the gastrointestinal tract of humans. Recently, variants of these bacteria have developed resistance to medically important chemotherapeutic agents. Multidrug resistant *Escherichia coli*, *Salmonella enterica*, *Vibrio cholerae*, *Enterobacter* spp., and *Staphylococcus aureus* are becoming increasingly recalcitrant to clinical treatment in human patients. Of the various bacterial resistance mechanisms against antimicrobial agents, multidrug efflux pumps comprise a major cause of multiple drug resistance. These multidrug efflux pump systems reside in the biological membrane of the bacteria and actively extrude antimicrobial agents from bacterial cells. This review article summarizes the evolution of these bacterial drug efflux pump systems from a molecular biological standpoint and provides a framework for future work aimed at reducing the conditions that foster dissemination of these multidrug resistant causative agents through human populations.

## 1. Introduction

### 1.1. Food Microbiology: Production and Spoilage

#### 1.1.1. Food Production

Safe food production is in high demand considering the global rise of foodborne illnesses and their frequent outbreaks. Contaminated foods cause serious public health concerns as more than 200 diseases have been known to be transmitted through contaminated food [1]. In 2007, the World Health Organization launched an initiative to estimate the global burden of foodborne diseases [2]. The major causative agents of these illnesses involve viruses, bacteria, parasites, toxins, metals, and prions. In particular, microorganisms, especially the bacteria, have become an important group of causative agents because most of their high frequencies of morbidity and mortality rates are related to foodborne illnesses [3]. Colonization of these bacteria in human beings can cause a broad spectrum of food borne illnesses such as bacteremia, meningitis, urinary tract infection, septicemia, wound infection, and central nervous system and gastrointestinal tract disturbances [4]. These diseases may occur as a result of infection, intoxication, or the reaction of the human body against pathogenic and opportunistic bacteria [5].

During the process of food production, the quality of foods can be affected by different factors, such as unhygienic preparation, storage, and unhealthy food animals. The risk of food contamination by enteric pathogens may occur on the farm when the soil is fertilized by human sewage or if crops are irrigated with sewage water. Moreover, food products are at risk of contamination with mishandling during processing and preparations where there are favorable conditions for pathogens [4]. Vegetables are frequently implicated in foodborne outbreaks, possibly due to farm contamination, rinsing harvested crops with unsafe water, cross contamination, and consuming raw or minimally cooked foods. Lack of safe (potable) water and refrigeration facilities, post production processes and poor personal hygiene of food handlers may also contribute to high levels of bacterial counts on foods, possibly placing consumer health at risk [6].

#### 1.1.2. Food Spoilage

Microorganisms can be considered to be the major cause of food spoilage although only a few of these organisms are responsible for offensive off-flavours [7]. Foods are dynamic systems where changes may occur in pH, atmospheric pressure, temperature, nutrient composition, and the microbiome over time, thus permitting spoilage bacteria to grow and produce spoilage metabolites. Each food product has its own unique flora, determined by the raw materials used, food processing parameters and subsequent storage and handling conditions.

The growth and metabolism of microorganisms are responsible for the degradation of organic and inorganic materials. Interaction (antagonism or symbiosis) between different microorganisms may influence these growth and metabolic systems [8]. This type of food degradation, known as food spoilage, causes changes in the sensory properties of food by the alteration of their chemical and physical natures. Such food alterations by bacterial contaminants are often the result of the production of amines, sulfides, alcohols, aldehydes, ketones, and organic acids, thus conferring upon foods unpleasant properties, such as discoloration, slime production, and off-flavours making the foods unacceptable for human consumption. The use of the term “unacceptable” often depends on the nature of the affected foods—for example, ammonia odors may be part of a desirable odor in some dried and fermented fish food products, but are often considered unacceptable in most other fresh and mildly preserved seafood products.

### 1.2. Bacterial Food Pathogens

#### Key Bacterial Pathogens, Virulence and Available Chemotherapy

Scallan and colleagues estimate that 37 million illnesses are acquired each year in the United States from food pathogens. An estimated 9 million of these pathogen-induced illnesses are domestically acquired [9]. Of these, 0.2 million are attributed to parasitic infections, 3.6 million to bacterial infections and 5.5 million to viral infections [9]. Key food-borne disease-causing bacteria include *Salmonella enterica, Staphylococcus aureus, Escherichia coli, Vibrio cholerae,* and *Enterobacter* spp.

##### *Salmonella* *enterica*

*Salmonella* species are facultative anaerobic Gram-negative rod-shaped bacteria classified under the family Enterobacteriaceae. These bacteria are motile, non-spore forming, and are part of the normal flora in the gut of vertebrates [10]. The modern nomenclature for this microorganism involves italicizing the genera and specific epithets, but not the serovars which are capitalized. *Salmonella enterica* serovar Typhimurium and *S. enterica* serovar Enteritidis cause worldwide epidemics of gastroenteritis, prominent of all human infectious diseases [11]. Consumption of contaminated chicken, beef, pork, sausage, meat paste, unpasteurized cheese, and lettuce is a major cause of food-borne salmonellosis [12]. The increasing incidence of antibiotic resistant clinical isolates of *S. enterica* enhances the risk of therapeutic failure in cases of life-threatening salmonellosis [13]. There are two types of *Salmonella* infections: the very well-known typhoidal *Salmonella* (caused by serotypes *S.* Typhi and *S.* Paratyphi A, B or C) and the less serious and generally self-limiting non-typhoidal *Salmonella* (caused by serotypes other than *S.* Typhi and *S.* Paratyphi). Both types of *Salmonella* infections are acquired by the fecal-oral transmission mode, but typhoid fever is closely associated with incidences direct human contamination of foods or sewage contamination of crops, meats, and water, and humans are the only known reservoirs of typhoidal *Salmonella*. Further, non-typhoidal *Salmonella* are widely distributed in nature and can be found in animal meat, poultry, eggs, seafood, *etc.* [14].

Chemotherapy used to treat *S. enterica* is less common since a study by Wiström and Norrby in 1995 found little to no benefit in the administration of fluoroquinolones [15]. Only early on, that is, within 48 h of the onset of symptoms, was there a benefit in taking norfloxacin [15]. It was once thought that providing antimicrobials to patients would shorten the duration of a *S. enterica* infection; however, nowadays, electrolyte and fluid replacement is the best treatment since the majority of infections are self-limiting [16]. In contrast, antibiotics are often indicated in patients who are severely ill, and the following chemotherapeutics are most often prescribed: the fluoroquinolones, trimethoprim sulfamethoxazole (TMP-SMZ), ampicillin, or extended-spectrum cephalosporins (e.g., ceftriaxone or cefixime). Regretfully, multidrug resistance has already been documented in a large number of *S.* Typhimurium isolates, particularly to TMP-SMZ and ampicillin [17].

##### *Staphylococcus* *aureus*

*Staphylococcus aureus* is a significant contributor to food contamination being the fourth largest cause of domestically acquired foodborne illnesses in the United States and affecting about 240,000 people annually [9]. *S. aureus* is a Gram-positive, non-spore forming, catalase positive, non-motile coccus which is ubiquitous in humans and in the environment, *i.e.*, soil, water and air. The ubiquitous nature of this microbe is no doubt attributable to its robust nature. *S. aureus* can survive under very dry conditions, under high osmotic pressure, in a wide range of pH conditions (4.5 to 9.3) as well as in a wide range of temperatures (6 °C to 48 °C) [18]. *S. aureus* causes gastroenteritis by production of heat-stable enterotoxins which induce staphyloenterotoxicosis or staphyloenterotoxemia within 1 to 7 h after consumption of contaminated food [18]. Affected humans will be ill for about a day and very rarely have serious complications other than dehydration associated with their symptoms of diarrhea and vomiting. There is an impressive array of virulence factors contributing to the pathogenesis of *S. aureus*, summarized by Murray, *et al.* [19] and Foster [20].

##### *Escherichia* *coli*

Pathogenic strains of *E. coli* bacteria are well known food pathogens acquired from traveling, best known as traveler’s diarrhea, but surprisingly not uncommon in the United States [21]. *E. coli* is a Gram-negative rod shaped bacterium belonging to the Enterobacteriaceae family, a large group of medically important microorganisms. The wild-type *E. coli* bacterium is a normal part of the gut microbiome of humans and other warm blooded animals. However, the enterovirulent *E. coli* are particularly problematic in human clinical medicine. Enterovirulent *E. coli* are distinctly named based upon the production of their associated virulence factors. There are six different *E. coli* pathotypes: enterotoxigenic *E. coli* (ETEC), enteropathogenic *E. coli* (EPEC), enterohemorrhagic *E. coli* (EHEC), enteroinvasive *E. coli* (EIEC), enteroaggregative *E. coli* (EAEC), and diffusely adherent *E. coli* (DAEC). In addition, each type has a different pathogenic scheme. The types of pathogenic *E. coli* most commonly involved in causing foodborne illnesses are ETEC, EPEC, EHEC, and EIEC [21]. Carcinogenic *E. coli* NC101 are discussed elsewhere [22].

ETEC is the causative agent of the illness commonly known as traveler’s diarrhea. The virulence factors that are most notable to this type are the heat-labile (LT) toxin and heat-stable (ST) toxins. ETEC can possess LT, ST, or both [23]. ETEC is generally a self-limiting infection but can resemble a cholera infection. Antibiotics are not required for treatment but do reduce the severity and duration of the symptoms. Mortality due to an infection with ETEC is seen mostly in children and kills about 380,000 a year in the world according to the World Health Organization [24]. Mortality rates in the US caused by ETEC are rare since these infections normally occur in places where poor sanitation is common [24]. Humans are the primary source of ETEC transmission and dissemination through populations via the consumption of contaminated water or food.

Members of the EPEC are identified as such if the locus for enterocyte effacement (LEE) is harbored on the pathogenicity island that is present in their genome. LEE encodes a 94 kDa outer-membrane protein called intimin [25]. EPEC was the first enterovirulent *E. coli* to be described, and it is now one of the best understood of the *E. coli* pathogens [26]. The infective dose of EPEC is lower in infants than in adults [27]. Thus, it is thought to be more virulent in infants and is usually connected to a relatively high infant mortality rate due to infantile diarrhea [27]. EPEC that are similar to ETEC are more common in underdeveloped countries where sanitary practices are less common [28]. Contamination of foods with EPEC are apparently sporadic but have been documented in mayonnaise, pickles, lettuce, raw beef, and raw chicken but can ultimately be any food that is subjected to fecal contamination [29].

A notorious group of potentially fatal *E. coli*, the EHEC, confer hemorrhagic colitis, bloody diarrhea, and hemolytic uremic syndrome (HUS) [30]. There are upwards of 400 serotypes of Shiga-toxin producing *E. coli* (STEC). The EHEC members are a subset of the STEC group [31]. Kidney failure, watery diarrhea progressing to bloody-diarrhea, blood clotting, low-grade fever, and vomiting are the general EHEC symptoms following exposure to contaminated food with an infective dose as little as 10 to 100 bacilli. Foods implicated in carriage of EHEC include ground meats, unpasteurized milk and fruit juice, lettuce, spinach, sprouts, and manufactured frozen cookie dough [32].

The Shiga toxin-producing *E. coli* non-O157 (STEC non-O157) is ranked sixth in the number of domestically acquired cases of food-borne disease annually in the United States with approximately 112,000 cases per year [31]. *E. coli* O111, O26, O121, O103, O145, and O45 are STEC non-O157 serotypes correlated with food borne illnesses [33]. Both STEC non-O157 and STEC O157 infections last approximately 8 days, and antibiotic chemotherapy is not always the best course of treatment since some reports have documented that killing of the STEC induces additional release of the Shiga toxins [31]. The major foods contaminated with STEC bacteria include beef and raw vegetables, and the mode of bacterial transmission can be person-to-person [34].

Enteroinvasive *E. coli* (EIEC) are one of the most publicized of the *E. coli* pathogens and account for the majority of *E. coli* outbreaks reported in the news media. This group of bacteria is similar to *Shigella* both biochemically and pathogenically, sharing many virulence factors [35]. Both pathogens cause bacillary dysentery, and EIEC is generally self-limiting when infecting otherwise healthy individuals [36]. Dysentery that is caused by EIEC is most often indistinguishable from other dysenteries caused by the other *E. coli* pathogens [37]. The reported mortality rate is zero, and the infective dose is 200 to 5000 cells (which is slightly higher than that of *Shigella*); the duration of the disease is approximately one week with symptoms of softened stool containing blood, vomiting, fever, chills, and abdominal cramps [19]. There are no specific foods which are linked to EIEC infections to date, and transmission may be person-to-person [19].

##### *Vibrio* *cholerae*

Both pathogenic and nonpathogenic species of *Vibrio* from the Vibrionaceae family live in marine and estuarine environments. All species of the *Vibrio* genus are Gram-negative, non-spore forming, rod-shaped bacteria with a slightly curved cellular morphology and a polar flagellum [19]. The pathogenic strains of *V. cholerae* cause the disease cholera and secrete the cholera toxin (CT), which in turn directly affects the human gastrointestinal tract leading to profuse watery diarrhea with a consistency known as the “rice water stool.” Cholera caused by virulent strains is diagnosed by symptoms of diarrhea and vomiting in humans. The classic distinguishing symptom of *V. cholerae*, *i.e.*, watery diarrhea, occurs in about 20% of infected individuals, and the so-called “rice water” stool occurs in approximately 20% of those presenting with watery diarrhea [38]. The disease is easily transmitted within humans by the consumption of contaminated water, or feeding on contaminated fish and crustaceans breeding in water contaminated by feces of individuals suffering from cholera [39]. Although the pathogenic species combined account for only approximately 15,000 cases of domestically acquired foodborne illness in the United States, on a world-wide scale the number of cases are between 3 and 5 million with periodic pandemics. Cases of cholera are usually seen in developing areas or largely poverty stricken areas, the majority being in Africa [19].

There is a 30%–50% mortality rate if rehydration is not administered after the onset of symptoms which can last from a few hours to 3 days after exposure to approximately 1 million bacterial cells [40]. Chemotherapy for the treatment of *V. cholerae* is not recommended as the primary source for the treatment in mild cases [41]. Apart from oral rehydration, antibiotics, such as doxycycline, are prescribed clinically for the treatment of severe cases of cholera, along with other antibiotics such as cotrimoxazole, erythromycin, tetracycline, chloramphenicol, furazolidone, and norfloxacin [42]. Doxycycline and tetracycline are used mostly for treatment of severe cases, along with large amounts rehydration and electrolyte therapy [19].

##### *Enterobacter* spp.

Species of *Enterobacter*, a Gram-negative rod and a member of the Enterobacteriaceae family of bacteria, can be found in diverse environments [43]. This microorganism can be acquired through endogenous and exogenous sources [43]. *Klebsiella* spp. can often be confused with *Enterobacter* spp., but a distinguishing factor is the motility of *Enterobacter* species [44]. Recent work has suggested that the *Enterobacter* core genome had originated from *Klebsiella* [45], probably the main factor for the confusion in distinguishing between the two organisms. *E. cloacae* and *E. aerogenes* are the most common members of the *Enterobacter* genus to cause infectious disease in humans [43]. Foods such as raw vegetables, dairy products, and raw shellfish are known to harbor these microbes [19].

Before the advent of widespread antibiotic usage, the *Enterobacter* species were rarely implicated as pathogens [46]. Since the 1970s these species have played a role in nosocomial infections [43]; there has been, however, little work conducted regarding their virulence factors that contribute to their pathogenicity. Instead, more focus has inadvertently been placed on their intrinsic resistance to antibiotics as well as their role in neonate infections since its resistance profile seems to be the primary cause of the observed increase in clinical prevalence [43,47,48].

### 1.3. Problem of Bacterial Resistance to Antimicrobial Agents

Although antibacterial agents are used to treat infectious disease caused by these types of pathogenic microorganisms, there is, nonetheless, a major public health concern due to the emergence, development and evolution of drug resistance mechanisms harbored by these pathogens and their variants. Continuous application of chemotherapeutic agents for treating pathogens, potentially results in the emergence of antibiotic resistant microorganisms due to selective pressures of antibiotic usage [49]. Emergence of single and multidrug resistant pathogenic microorganism due to extensive use of antibiotics for clinical treatments eventually result in failure of treatments due to resistance mechanisms developed by these and other pathogens. Bacterial cells acquire resistance through several molecular and cellular systems [50,51,52]. Multidrug resistance (MDR) phenotypes, observed in various bacterial organisms, are often selected as a result of single antibiotic exposure [53]. The wide spread use and prescription of antibiotics has resulted in increased antibiotic resistance in bacteria [53]. Many patients do not complete their full course of prescribed antibiotics, giving the bacteria a chance to survive and become adapted to an environment with low concentrations of antibiotics. This leads to development of antibiotic resistance in the long run [54].

## 2. Mechanisms of Bacterial Drug Resistance

**Figure 1:**
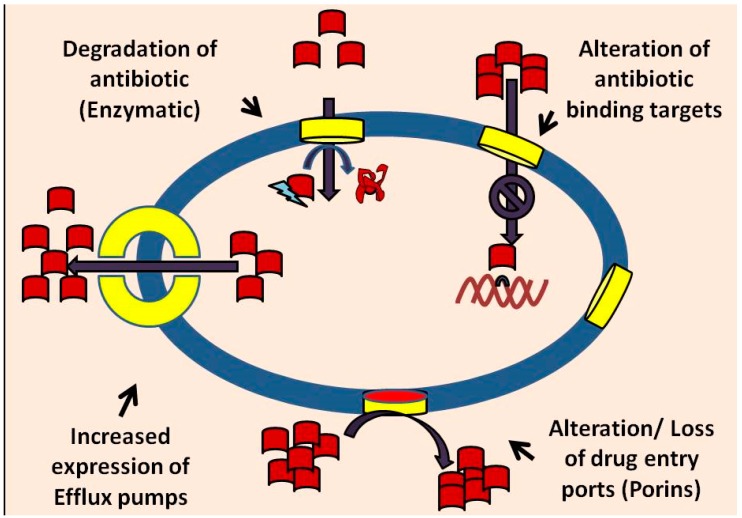
Bacterial antibiotic resistance mechanisms. Red blocks indicate antibiotics. Yellow channels indicate drug entry ports/porins. Mechanisms of bacterial resistance include the alteration of drug binding targets (DNA); degradation of antibiotics by enzymatic action; expression of efflux pumps on the cell membrane; altered or loss of porin/drug entry ports, the latter two mechanisms of which thereby reduce the intracellular concentration and permeability of the drug into the cell, respectively. This figure was adapted from Kumar and Varela, 2013 [52].

Drug and multidrug resistance mechanisms have recently been considered as virulence factors in pathogenic bacteria [55]. The mechanisms responsible for these increased antimicrobial resistances include biofilm formation, alteration of binding sites, enzymes that can inactivate antibiotics, decreased membrane permeability and active efflux of antimicrobials [52,53] (Figure 1). These various drug resistance mechanisms are considered briefly below.

### 2.1. Biofilms

One of the mechanisms of bacterial drug resistance includes biofilm production. Conventional resistance mechanisms, such as up-regulated efflux pumps and mutations in antibiotic target molecules in bacteria also contribute to the survival of biofilms [56]. Several mechanisms are reportedly responsible for the antimicrobial resistance in biofilm structures: (*i*) Poor diffusion of antibiotics through the biofilm polysaccharide matrix, although some antibiotics are able to penetrate the matrix [57]; (*ii*) physiological changes due to slow growth rates and starvation responses, such as nutrient deprivation or environmental stress [58]; (*iii*) phenotypic changes in biofilm-forming bacteria [59]; (*iv*) quorum-sensing [56]; (*v*) the expression of efflux pumps [60]; and (*vi*) persister bacterial cells, which resist killing when exposed to antimicrobials [61]. Biofilms constitute a crucial challenge in clinical medicine with respect to antibacterial resistance [62].

### 2.2. Drug Target Modification

The widespread overuse and misuse of antibiotics create consequence of antibiotic resistance. Among many, bacteria have various strategies to inhibit cytotoxic effects of antibiotics. One of the main strategies is the alteration of the target sites (sites of action) of antibiotics, often by mutation that alters the drug binding site, essentially inactivating the antibiotics and allowing the bacterial pathogen to grow in the presence of the antimicrobial agent [52]. Altered cellular drug targets include, for example, DNA gyrase, a target of quinolones and fluoroquinolones [63], RNA polymerase, a drug target of rifampin and related antimicrobials [64], bacterial ribosomes, which are the major sites of antibiotic action in bacterial cells and are targets of tetracycline and other protein synthesis inhibitors [65,66].

### 2.3. Enzymatic Degradation of Antimicrobial Agents

Enzymatic degradation of antimicrobial drugs is a common biological processes which confers bacterial resistance to clinically-used antibiotics [67]. Most of the antibiotics have hydrolytically susceptible chemical bonds (e.g., esters and amides); and several enzymes have evolved that cleave these vulnerable bonds and as a result produce an inactive form of the antimicrobial agent thus providing a means for the bacteria to grow. Amidases can cleave the beta lactam rings of the penicillin and cephalosporin classes of drugs [68]. The acetyltransferase enzymes, such as the aminoglycoside acetyltransferases, chloramphenicol acetyltransferases, and streptogramin acetyltransferases, are the largest family of drug resistance enzymes [69]. These enzymes have the potential to modify antibiotics and play an important role in structural alterations which impair the target binding sites. Other covalent modification strategies include, for instance, phosphorylation by aminoglycoside kinases [70], nucleotidylation of lincosamide antibiotics [71], ADP-ribosylation of rifampin [72], and glycosylation of macrolides [73].

### 2.4. Reduced Drug Permeability across the Bacterial Membrane

Reduced membrane permeability to chemotherapeutic agents is an important resistance mechanism in which the antibiotics are unable to enter into the cytoplasm of bacterial cells where drug target sites are located [74]. Mechanisms that are involved include modifications of the outer membrane lipid barrier and porin mediated permeability, two important antibiotic resistance properties. The outer membrane in Gram negative bacteria consists of phospholipid and lipopolysaccharide (LPS). LPS is composed of lipid A, a core oligosaccharide and the *O*-antigen [75]. LPS acts as a strong permeability barrier in these Gram negative bacteria such as *Pseudomonas aeruginosa*, *V. cholerae* and *S. enterica*; thus these bacteria are intrinsically resistant to many macrolide antibiotics such as erythromycin and azithromycin, plus novobiocin, rifamycin, *etc.* [76].

A second mechanism in this category of reduced drug permeability and resistance involves outer membrane channel proteins, such as porins which normally allow antimicrobial agents to enter into the cells [77]. Two major porin-based mechanisms for bacterial antibiotic resistance include (i) alteration of the outer membrane involving the loss, reduction or replacement of porins; and (ii) alteration in the functional activities of porins due to specific mutations [77]. Microorganisms such as *E. coli*, *P. aeruginosa*, *Neisseria gonorrhoeae*, *Enterobacter aerogenes* and *Klebsiella pneumoniae* acquire antibiotic resistance through the loss or functional changes in porins [78], thus acquiring resistance to carbapenems, tetracycline, fluoroquinolones, aminoglycosides and chloramphenicol [79].

### 2.5. Active Efflux of Drugs from Bacterial Pathogens

**Figure 2:**
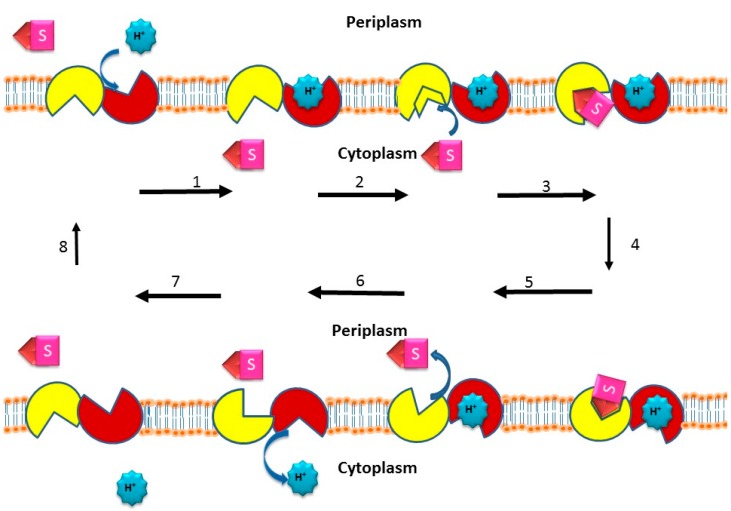
Drug/H^+^ efflux pump transport mechanism. Starting with an empty pump in which the H^+^ binding site faces the outside or periplasm and the drug (*i.e*., substrate) binding site faces the inside or cytoplasm, the drug / proton antiport cycle is as follows: *(step **1**)* The H^+^ binds the outside face of the empty efflux pump; *(step **2**)* the binding affinity of the pump for the drug substrate increases on the cytoplasmic side; *(step **3**)* the drug binds the inside face of the pump; *(step **4**)* a conformational change occurs such that the drug and H^+^ binding sites switch sides, *i.e.*, an alternating access mechanism [81] thus essentially translocating both drug and H^+^ through the pump and across the membrane in opposite directions—the bound drug consequently faces the outside or periplasm, and the bound H^+^ faces the cytoplasm; *(step **5**)* the drug is released to the outside or periplasm; *(step **6**)* the H^+^ is then released into the cytoplasm; *(step **7**)* the efflux pump then reorients itself so that the drug binding site now faces the cytoplasm, and the H^+^ binding site faces the outside or periplasm; *(step **8**)* the empty efflux pump is then ready to begin another drug/H^+^ antiport cycle. The two α-helical bundles representing the two-fold rotational axis of symmetry during transport [82] are shown in yellow and red. The drug substrate is denoted as S. The protons are blue. The proton-driven drug efflux pump mechanism was adapted from references [83,84,85].

Pathogenic bacteria that are causative agents of infectious diseases are a major concern in clinical studies because of their ability to confer single and multidrug resistance to antibacterial agents. Unfortunately, the excessive use of antibiotics to treat infectious diseases has resulted in the development of drug and multidrug resistance in these “superbugs.” Bacteria show resistance to antibacterial agents and other toxic compounds by a mechanism known as active efflux, where the integral membrane transporters known as drug efflux pumps, prevent the accumulation of drugs inside the bacterial cells (Figure 2). Based on the source of energy and amino acid sequence, the bacterial efflux transporters are classified into five different superfamilies (i) the major facilitator superfamily (MFS); (ii) the small multi-drug resistant superfamily (SMR); (iii) the multi antimicrobial extrusion protein superfamily (MATE); (iv) the ATP-binding cassette superfamily (ABC); and (v) the resistance-nodulation-cell division superfamily (RND) [51,52,80].

#### 2.5.1. Discovery of Single and Multidrug Efflux Pumps

Antibiotic efflux as a mechanism for bacterial resistance to antimicrobial agents was first discovered and studied physiologically in the laboratory of Levy [86,87,88]. This seminal discovery demonstrated that the antimicrobial agent tetracycline, an antibiotic, was actively extruded from the bacterial cell thus diluting the intracellular concentration of the inhibitory agent from the cytoplasm where the target of the drug, namely, the ribosome, resides and therefore confers tetracycline resistance upon the bacterium [89]. Subsequently, it was found that the tetracycline efflux pumps were driven by a proton motive force as predicted by Mitchell [90,91] in which the proton gradient generated by the respiratory chain moved protons across the biological membrane down their ion concentration gradient in order to actively drive the exit of the drug from the cell [87,88]; this solute translocation process across the membrane is known as secondary active transport, or active drug efflux [83,84,89]. One of the first key multidrug efflux pumps to be discovered, Bmr, was found in the microorganism *Bacillus subtilis* [92]. This transporter conferred bacterial resistance to multiple structurally different antimicrobial agents, such as chloramphenicol, puromycin, ethidium bromide, rhodamine, and tetraphenylphosphonium [92]. Interestingly, the Bmr efflux pump showed homology to the *S. aureus* norfloxacin efflux pump, NorA, which at the time was considered a single drug transporter [93]. The homology shared between Bmr and NorA, however, predicted that NorA also had multiple substrates, which when tested physiologically was definitively demonstrated [94]. Since then, both pathogenic and non-pathogenic bacteria have been found to harbor and effectively express multidrug efflux pumps, and many of these transporters belong to the MFS [80,95]. Therefore, the solute transporters that comprise the MFS are important not only from a fundamental molecular biological perspective but from a biomedical one, as well.

#### 2.5.2. Efflux Pumps of *Staphylococcus aureus*

##### QacA and QacB Multidrug Efflux Pumps

Qac A/B along with NorA are probably the best characterized efflux pumps in *S. aureus*. QacA and QacB are members of MFS, and both transporters rely on the proton motive force for drug extrusion from the bacterial cell [96]. QacA and QacB are both 514 amino acids in length making each a predicted 55 kDa protein [97]; and gene expression of both *qacA* and *qacB* is regulated by QacR, a substrate responsive regulator, reviewed in reference [98]. QacA was linked to resistance against acriflavine, ethidium bromide, quaternary ammonium compounds (QACs), propamidine isethionate, and diamidinodiphenylamine dihydrochloride encoded on plasmid pSK1 [99]. The *qacB* determinant was first detected in the early 1950s on DNA plasmids and particularly on plasmid pSK23 [100]. The substrate profile of QacA includes a wide range of dyes, QACs, biguanidines, diamidines and guanyl hydrazones [98]. QacA was the first 14 α-helical transmembrane antimicrobial transport protein to be studied by using fusion proteins with alkaline phosphatase and β-galactosidase reporter enzymes [100]. The *qacA* and *qacB* genes are similar in their DNA sequences, differing by only 7 nucleotides [100]; thus, it is not surprising that their substrates are similar but not identical. For example, QacB extrudes monovalent cationic dyes and QACs but has little to no ability to extrude divalent cationic drugs, diamidines, and biguanidines [98]. Acidic residues in QacA are important for extrusion of divalent cations [101]. The difference in substrate profiles between QacA and QacB can be attributed to the lack of an aspartate residue (D) at position 323 in the transmembrane segment (TMS) 10 of the QacB polypeptide [100]. Evolutionary analysis suggests that QacA has evolved from QacB [98]. In a recent study with *S. aureus* clinical isolates there was an association with *qacA* and *qacB* presence in MRSA (8% of MRSA strains harbored the *qac* genes) as opposed to MSSA isolates with only 3.3% harboring *qacA* and *qacB* [102]. Moreover, Otter and colleagues found that carriage of *qacA* produces a selective advantage for *S. aureus* when cleaning with chlorhexidine [103]. These findings were supported by Shamsudin and colleagues who observed resistance to chlorhexidine and benzethonium chloride in MRSA isolates carrying *qacA/B* [104] as well as in a study of *qacA/B* in hospital acquired-MRSA isolates [105].

Regulation of expression for these two efflux proteins is accomplished by QacR [106]. The *qacR* gene is upstream of both *qacA* and *qacB* and belongs to the TetR family of repressors [97]. QacR regulates the transcription of the transcripts encoding the efflux proteins by binding to the large inverted repeat (IR1) sections that are located downstream from the promoter of the *qac* genes [107]. QacR binds IR1 with its DNA binding helix-turn-helix motif. This IR1 element overlaps the transcription initiation site for *qacA/B* [107]*.* The repressor binds in an unconventional manner compared to the normal TetR repressor proteins because it cooperatively binds two dimers not using protein-protein interactions [108]. Once the first QacR protein binds the DNA element, it widens the DNA allowing for the second dimer to bind [108]. Once the repressor is bound to IR1, the presence of QacA/B substrates induces a conformational change to QacR through the QacA/B substrate binding directly to QacR; hence, the repressor falls off the DNA and allows transcription from the *qac* genes [109]. This regulation has been demonstrated to proceed in a substrate concentration-dependent manner [106]. Interestingly several substrates of the QacA efflux pump do not bind the QacR repressor and will not, therefore, induce the conformational change required for the repressor to fall off the DNA. Therefore, QacR is suggested to be a weak repressor and that there is a basal level of *qacA* expression, recently reviewed in reference [110].

##### LmrS Multidrug Efflux Pump

LmrS is a 480 amino acid proton-coupled multidrug antiporter belonging to major facilitator superfamily (MFS). The *lmrS* gene is 1443 nucleotides long and is present on the *S. aureus* chromosome. LmrS has a 39% identity with the lincomycin resistance protein LmrB from *B. subtilis* and 25% identity with the efflux pumps EmrB of *E. coli* and FarB of *N. gonorrhoeae* [111]. The 47 kDa predicted protein product is composed of 14 transmembrane α-helices that reside in the plasma membrane of *S. aureus* to extrude antimicrobials. This efflux pump efficiently extrudes lincomycin, kanamycin, linezolid, and fusidic acid [111]. The regulator of LmrS has not yet been determined.

##### MdeA Multidrug Efflux Pump

The 1437 bp *mdeA* gene is located on the bacterial chromosome of *S. aureus* and is predicted to encode a multidrug efflux pump, MdeA, with 479 amino acid residues [112]. Its substrates are tetraphenylphosphonium chloride (TPPCl) (showed an 8-fold increase in MIC values as compared to control cells) and norfloxacin (resulted in a 2-fold increase when compared to control cells) and Hoechst 33342 (16-fold increase as compared to control cells). Fluoroquinolones were initially shown not to be substrates for MdeA by Huang and colleagues in 2004, but in a study by Yamada and colleagues in 2006, there was evidence that fluoroquinolones were moderate substrates [113]. The ~52 kDa membrane bound protein has 14 predicted transmembrane domains and belongs to the major facilitator superfamily [112]. MdeA has a 23% identity to QacA of *S. aureus.* Other pumps with similarity to MdeA include LmrB of *Bacillus subtilis*, EmrB of *E. coli*, *and* FarB of *N. gonorrhoeae* [112]*.* After experimentation with reserpine and carbonyl cyanide *m-*chlorophenylhydrazone (CCCP), the data suggested that the energy source for MdeA multidrug efflux is through the utilization of the proton motive force [112]. The expression of efflux pumps can be due to spontaneous induction for the pump’s regulators or through spontaneous mutations in the pump to stabilize them in the membrane. The promoter region of the *mdeA* gene was sequenced and two point mutations were found; one mutation in the −10 region (C→A) and the second mutation in the −35 region (G→T) [114]. Similar mutations had also been reported by Huang and colleagues in 2004, where a >300 fold increase in *mdeA* transcription was observed [112]. Likewise, in a study by DeMarco and colleagues in 2007, the point mutation transversion (G→T) in the −35 region was observed in two strains, MRSA252 and *S. aureus* RF122 [115]. In that same study, blood isolates of *S. aureus* were collected, and the susceptibility profiles were determined followed by efflux pump expression analysis; approximately 49% of the studied *S. aureus* strains had overexpressed efflux pumps [115]. About half of the 49% consisted of overexpressed MepA, MdeA, and/or NorABC [115]. MdeA overexpressing strains accounted for 4% of the 114 strains collected and had between 3 and 6 substrates [115]. This same study pointed out the point mutation (G→T) of MRSA252 and *S. aureus* RF122 in the −35 region. The regulators of this efflux protein have not yet been identified.

##### QacG, QacH, and QacJ Multidrug Efflux Pumps

Other characterized multidrug efflux proteins in *S. aureus* include QacG, QacH, and QacJ. All of which are active antiporters of Qacs and dyes, are encoded on plasmids, and belong to the SMR family [116]. QacG, first isolated from the pST94 plasmid, is 107 amino acids in length and differs from Smr by only 33 amino acids dispersed throughout the peptide chain resulting in a 2% identity to the Smr protein [117]. QacG has four transmembrane segments [117]. QacH was first identified in 1998 by Heir and colleagues on the p2H6 plasmid [118]. This QacH efflux pump is 107 amino acids in length, and the *qacH* gene shows a 76% nucleotide identity to *smr* and 70% nucleotide identity to *qacG*. QacJ was first identified in 2003 on pNVH01 plasmid [119]. The primary amino acid sequences of QacJ, QacG and QacH are similar to each other. For example, QacJ is also 107 amino acids long and is 6% similar to QacG, 4% similar to QacH, and 5% similar to Smr [119]. The regulatory element(s) for all of these pumps are not yet identified. In a 2008 study by Correa and colleagues, 21 isolates of *S. haemolyticus* were isolated over a period of ten years. The following genes encoding efflux pumps were found aiding these isolates in drug resistance: *smr* in all of the isolates, *qacG* in 11 isolates, *qacH* in 10 isolates and *qacJ* in 4 isolates [120].

##### NorA Multidrug Efflux Pump

NorA is one of the best-studied multidrug efflux pumps of *S. aureus*. In 1986, a chromosomally encoded gene, *norA*, was first observed in a fluoroquinolone-resistant isolate from a Japanese hospital [121]. The DNA sequencing of the resistance-conferring genetic element in 1990 predicted that NorA consists of 388 amino acids (45% are hydrophobic), with a molecular weight of 42,265 Da and 12 hydrophobic membrane spanning regions [122]. The NorA efflux pump was found to be similar to the multidrug efflux pump of *B. subtilis*, Bmr (44%) and partly with the closely-related tetracycline efflux transporters, denoted as TetA [94].

NorA can extrude a wide range of antimicrobial agents like fluoroquinolones and structurally distinct non-fluoroquinolone agents [94]. The proton motive force is used for the transport of antimicrobial compounds across the cell membrane by a vectorial process known as the H^+^: drug antiport mechanism [123]. The increase in resistance is due to the increased expression of the *norA* gene [124] which may be mediated by a regulatory protein or by the mutation in the promoter region of *norA* [125] or may also be due to the transcription of *norA* gene [123].

As drug efflux is a resistance mechanism for the efflux of antimicrobials, inhibitors are also present that will inhibit the drug efflux system. Examples include CCCP and nigericin which dissipates the proton gradient of the membrane resulting in inhibition of NorA-mediated norfloxacin transport [124]. Reserpine also inhibits NorA function by an undefined mechanism [126]. Other inhibitors of the NorA efflux pump includes chalcone [127], capsaicin [128], kaempferol rhamnoside [129], ofloxacin based efflux pump inhibitors [130], thiopyranopyridine moiety [131], omeprazole derivatives [132], flavones, isoflavones, neohesperidosides, pentaester, spinosan A, pterocarpan, orizabin XIX, orizabin IX, epigallocatechin gallate, epicatechin gallate, coumarin epoxide derivative, bergamottin epoxide derivative and piperidine alkaloids [133].

The regulation of *norA* is by MgrA, a member of a MarR group of transcriptional regulators [51]. MgrA is a negative regulator of *norA* as expression of MgrA is associated with a 2.3-fold reduction in the synthesis of *norA* transcript [126,134]. The phosphorylated form *i.e*., MgrA-P gets released from the *norA* promoter, causing the transcription of *norA* [135]. Phosphoryation of MgrA, MgrA-P, is performed by the serine/theorine kinase PKnB [136] and the dephosphorylation of MgrA-P is accomplished by RsbU [135]. MgrA, also affects other pumps like NorB, NorC and Tet38 [137,138] resulting in decreased resistance to hydrophilic (norfloxacin and ciprofloxacin) and hydrophobic (moxifloxacin and sparfloxacin) quinolones, tetracycline and chemical compounds like ethidium bromide, cetrimide and tetraphenylphosphonium (TPP) [137]. NorG, a transcriptional regulator from the GntR-like (gluconate regulatory protein) family, can also bind with the promoter of *norA* [137]. NorA can also be modulated by the two component regulatory system, ArlR-ArlS, which can control autolysis rate, attachment to a polymer, proteolytic activity, cell growth and pathogenesis [134,139]. Other modulators include ferric uptake regulator (*fur*), which acts as a positive regulator of *norA*. Besides this, NorA is also involved in secretion, recycling, and export of siderophores from *S. aureus* [134].

##### NorB Multidrug Efflux Pump

NorB is a chromosomally encoded efflux pump that contains 463 residues with 12 transmembrane segments [140]. The *norB* gene is 1392 bp long and encodes a 49 kDa protein. Belonging to the major facilitator superfamily of efflux pumps, NorB shares 30% sequence similarity with NorA of *S. aureus*, 30% with Bmr, 41% with the Blt transporter of *B. subtilis* and 39% with QacA of *S. aureus* [140]. Expression of *norB* confers resistance to not only NorA substrates such as hydrophilic fluoroquinolones (for example, norfloxacin and ciprofloxacin), biocides (e.g., tetraphenylphosphonium and cetrimide) and dye (ethidium bromide), but also shows resistance to other non-NorA substrates like hydrophobic fluoroquinolones (moxifloxacin and sparfloxacin) and also to tetracycline at a lesser level [110]. However, addition of reserpine led to a decrease in drug resistance mediated by NorB [140].

In another study, NorB was found to be associated with reduced response to antibacterial agents in an abscess environment [141]. Expression of *norB* can be increased up to 8-fold when there is a shift in pH from 7.0 to 4.5 causing increased resistance to moxifloxacin [142]. NorB transcript level can also be increased with reduced oxygen level [143]. A model for this regulation was postulated by Hooper and colleagues in which under reduced aeration conditions serine protease cleavage of the MgrA monomer prevents its formation to a dimer, thus preventing its binding to the *norB* promoter resulting in an increase in the syntheses of the *norB* transcript and NorB and a consequent reduction in moxifloxacin susceptibility [143].

Both MgrA and NorG are involved in its regulation. MgrA-P which is released from the promoter of *norA* following phosphorylation, binds promoter of *norB* causing repression of *norB* [110]. Under acidic stress, decreased level of MgrA-P may result from reduced level of PKnB causing increased expression and decreased repression of *norB* [142]. NorG, can not only bind to the promoter of *norB* but its expression can cause 3-fold increase in *norB* expression, with 4-fold increase in the level of resistance to quinolones indicating that *norB* can be activated by NorG [137]. However, MgrA acts opposite to NorG by being an indirect repressor for *norB* [137].

##### NorC Multidrug Efflux Pump

NorC is a chromosomally encoded efflux pump consisting of 462 amino acid and 14 transmembrane segments [144]. It belongs to the major facilitator superfamily with 61% amino acid similarity with NorB [144]. NorC confers resistance to quinolones such as ciprofloxacin, norfloxacin, sparfloxacin, moxifloxacin, garenoxacin and to the dye rhodamine 6G [110]. NorC is also negatively regulated by MgrA [144]. NorG also binds to the putative promoter of *norC* [137] and affects negatively the expression of *norC* [110].

##### NorD Multidrug Efflux Pump

NorD is a more recently studied chromosomally encoded efflux pump of the MFS, consisting of 12 membrane-spanning domains. Its expression is upregulated in subcutaneous abscess. The substrates of NorD are not known but the expression of *norD* doesn’t change susceptibility to various antimicrobial agents like quinolones (ciprofloxacin, norfloxacin, moxifloxacin, delafloxacin, and levofloxacin), tetracycline, polymyxin B, daptomycin, nalidixic acid, trimethoprim, ethidium bromide or triclosan. A 3-fold decrease in *norD* expression is seen with a shift to an acidic environment (pH 5.5). Although free iron restriction upregulates *norD* expression, Fur is a negative regulator of *norD* expression [145].

##### TetA(K) Tetracycline Efflux Pump

TetA(K), or Tet(K), is a plasmid-encoded efflux pump that acts as an Na^+^(K^+^) / H^+^ antiporter. It consists of 459 amino acids [146] having a similarity of 60% with TetA(L) [147]. TetA(K), a 7 kDa protein consisting of 14 transmembrane segments, belongs to the MFS of transporters [148]. TetA(K) confers high levels of resistance to tetracycline, oxytetracycline and chlortetracycline. However, less resistance is seen for antibiotics like minocycline, 6-demethyl-6-deoxytetracycline and doxycycline. TetA(K) does not extrude these particular tetracyclines due to the lack of a hydroxyl substituent at the 6th position of these antibiotics. This may be the reason why minocycline is effective against *S. aureus* carrying TetA(K), as this transporter is unable to pump out this substrate from the cytoplasm of the bacterium [149]. The bacteria harboring TetA(K) are also resistant to other external stresses, like Na^+^ stress, alkali stress and K^+^ insufficiency stress [147]. When Glu-397, which resides in helix 13, was altered with another amino acid residue, the transport activity for tetracycline was consequently completely lost [150]. Various natural compounds modulate TetA(K), including carnosic acid, carnosol, lamiaceae, epigallocatechin gallate and diterpenes [151,152,153].

##### Tet38 Tetracycline Efflux Pump

Tet38 is a chromosomally encoded drug efflux pump which is associated with resistance to tetracycline [140,141]. Tet38 is an MFS efflux pump that shares 46% similarity with the plasmid encoded TetA(K) of *S. aureus* and 45% similarity with TetA(L) of *B. subtilis* [140,154]. The *tet38* gene encodes an integral membrane protein that is predicted to contain 450 amino acid residues with 14 predicted transmembrane domains. The gene is 1353 bp long, encoding a 19 kDa protein [140]. Expression from the *tet38* gene of Tet38 leads to a 32-fold increase in the resistance to tetracycline but not for other drugs including minocycline [140]. The *tet38* gene is negatively regulated by MgrA [140], but NorG does not appear to bind the control elements for *tet38* [110].

##### SdrM Multidrug Efflux Pump

SdrM is a multidrug efflux pump with 14 predicted transmembrane segments and belongs to MFS. SdrM is 23% identical and 68% similar to NorB; likewise, SdrM is 21% identical and 63% similar to QacA [155]. In this same study it was found that MgrA also regulates its expression, and mutation of the *mgrA* gene may cause enhanced expression from the *sdrM* (*Staphylococcus* drug resistance) gene leading to the emergence of more resistant strains. Low level of resistances to antimicrobial agents like acriflavine, ethidium bromide, fluoroquinolone, and norfloxacin are observed, supporting the notion that SdrM is an energy-dependent multidrug efflux pump [155].

##### Mef(A) Multidrug Efflux Pump

The Mef(A) efflux pump was first identified in *Streptococcus pyogenes* in 1996 [156]. Mef(A) is a chromosomally encoded multidrug efflux pump and a member of the MFS, reviewed in reference [157], and the gene is predicted to encode a largely hydrophobic 2 kDa protein with 90% similarity to *mefE* [158]. The first description of the *mef(A)* gene from *S. aureus* was reported in 2002 [159]. The *mef(A)* genes are widely distributed among other Gram-positive bacteria such, as *S. pneumoniae*, Group C and G Streptococci, *Corynebacterium*, *Micrococcus* and *Enterococcus* [160], plus Gram-negative bacteria such as *N. gonorrhoeae*, *Bacteroides*, *Pantoeae*, *Citrobacter*, *Enterobacter*, *Escherichia*, *Klebsiella*, *Morganella*, *Proteus*, *Providencia*, *Pseudomonas*, *Ralstonia*, *Serratia*, *Stenotrophomonas* and *Acinetobacter* [161]. Mef transporters can actively efflux macrolides but cannot extrude lincosamides and streptogramins; thus, cells harboring Mef are also referred to as having the M phenotype [162].

#### 2.5.3. Efflux Pumps from *Escherichia coli*

##### The AcrA, AcrB, and TolC Multidrug Efflux Pump

The AcrAB-TolC efflux pump consists of the outer-membrane channel TolC, the secondary active transporter, AcrB, located in the inner membrane, and the periplasmic protein, AcrA, which bridges these two integral membrane proteins [163]. This transporter system has the ability to allow vectorial transport of different chemical compounds, conferring a profound drug resistance profile upon *E. coli* to a broad range of antibiotics. According to a genetic and structural study of the AcrAB-TolC multidrug efflux pump, a stable, active, and complete pump depends on the relationship of the AcrB β-hairpins, belonging to the DN (distal amino) and DC (distal carboxy) subdomains, with TolC [164]. The study demonstrated a drug hypersensitivity phenotype due to a failure of an AcrB DN β-hairpin deletion mutant to engage TolC, leading to compensatory alterations in the lipoyl and β-barrel domains of AcrA. Furthermore, mutations in AcrA showed a failure to interact with TolC, inducing TolC to open up for drug expulsion [164]. Based on a study by Sato and colleagues of drug resistant *E. coli* in dogs and humans, lower levels of intracellular enrofloxacin coincided with higher expression of AcrA, and the AcrAB-TolC efflux pump showed a high level of fluoroquinolone resistance *in vitro* [165]. This result suggested that the AcrAB-TolC efflux pump confers resistance against fluoroquinolone and other antimicrobials [166].

##### EmrE and SugE Multidrug Efflux Pumps

EmrE and SugE are members of the small multidrug resistance family (SMR), the members of which consist of 110 amino acids in length, making them the smallest multidrug resistance transporter proteins known [167]. The EmrE and SugE transporters of *E. coli* share 27% sequence identity and 52% sequence similarity [166]. The EmrE transporter, also known as the *Escherichia* multidrug resistance protein, appears to be one of the most extensively characterized proteins in the SMR family and is responsible for conferring resistance to lipophilic cations including DNA intercalating dyes and quaternary ammonium compounds [167]. Because of its small size, it is hypothesized that EmrE functions as a homo-oligomer, which in turn is driven by the influx of protons across the plasma membrane, leading to a subsequent efflux of multiple drug substrates [167]. EmrE is believed to interact with a wide range of structurally distinct substrates, suggesting that the substrate binding pocket is extremely flexible or that multiple substrate binding sites exist [167]. SugE, on the other hand, is defined to have poor activity in which it does not transport drugs that are commonly transported by other multidrug resistance proteins, such as EmrE [166]. In fact, it seems that SugE would promote drug sensitivity rather than drug resistance [166]. The genetic determinant for SugE was initially identified as a gene that suppresses a *groEL* mutation and mimics the effect of GroE expression of the *Klebsiella pneumonia*e nitrogenase activity when expressed on a multi-copy plasmid [166,167]. These findings suggested that SugE could function as a drug importer [168].

##### MdfA Multidrug Efflux Pump

Although the MFS efflux pumps of *E. coli* were first recognized before 1993, there were no indications at that time that would have predicted the enormous impact the MFS itself would have on the field of solute transporter biochemistry and molecular biology [169]. However, within several years of intense research after its discovery, early investigators showed that the MFS could be expanded from 12 to a total of 74 families [169]. The MFS consists of uniporters, symporters, and antiporters [169]. Each of the families within the MFS are responsible for the transport of a distinct set of related and unrelated substances, including simple monosaccharides, oligosaccharides, amino acids, peptides, vitamins, enzyme cofactors, drugs, chromophores, nucleobases, nucleosides, nucleotides, iron chelates, and organic and inorganic anions, and cations [169]. There is presently no functional evidence of activity for 17 members within the MFS families from *E. coli* [169].

According to Li and Nikaido, several transporters of the MFS from *E. coli* carry out their solute transport functions by using MFP and OM components as multi-protein pumps, such as the EmrAB-TolC multidrug efflux pump, reviewed in reference [170]. MdfA confers resistance to chloramphenicol, a substance that is generally inactivated by a specific chloramphenicol acetlytransferase enzyme (CAT), encoded by a plasmid gene. MdfA, also known as CmlA or Cmr, is a chromosomally-encoded MFS transporter [171]. It is a multidrug pump that has, however, a limited substrate range [171]. Recently, investigators isolated the *flo* gene located on a large plasmid from a florfenicol- and chloramphenicol-resistant *E. coli* isolate that was associated with bovine diarrhea, suggesting a widespread distribution for this determinant through animal populations [172].

##### MacA and MacB Multidrug Efflux Pumps

MacA and MacB are ABC-type (ATP-binding cassette) efflux pumps, which are driven by ATP hydrolysis and play an important role in mediating resistance against antibiotics when the determinants for the major efflux pumps, *acrAB*, are deleted in *E. coli* [173]. In mammalian systems, the ABC transporters are the leading drug efflux transporters responsible for multi-drug resistance in cancer cells [174]. These primary active transporters are also responsible for the uptake of a wide range of molecules, plus the export of proteins and toxic metal ions (e.g., arsenite) from bacteria [174]. The precise number of functional ABC transporters in *E. coli* K12 serotype is still uncertain even with the sequencing of the *E. coli* K-12 genome and characterization of many of the ABC transporters genes encoded therein. There are over 70 discrete ABC transporters including putative ABC transporters that have been identified by several scientific reports. Moreover, the uropathogenic *E. coli* strain CFT073 and O157:H7 strain Sakai are known to have 83 and 82 ABC transporter gene systems, respectively [175,176].

#### 2.5.4. Efflux Pumps from *Vibrio cholerae*

##### EmrD-3 Multidrug Efflux Pump

EmrD-3 from *V. cholerae*, which was cloned in our laboratory, is a multidrug efflux pump that belongs to the major facilitator superfamily (MFS) [177]. EmrD-3 is predicted to consist of 12-transmembrane helices with their *N*- and *C*-termini residing in the cytoplasmic side of the inner membrane. The presence of EmrD-3 in a host bacterium prevents the accumulation inside of the cell of certain chemotherapeutics, like erythromycin, rifampin, and chloramphenicol; furthermore, EmrD-3, like LmrS from *S. aureus*, enables transport of and resistance towards the fluorescent dye ethidium bromide [177]*.* EmrD-2 from *V. cholerae* O395 (NCBI Reference: NC_0121), and EmrD-1 from *V. cholerae* M66-2 (NCBI Reference: NC_0121), also belong to the MFS, but both determinants of which are uncharacterized physiologically as of this writing [178].

##### VceB Multidrug Efflux Pump

Another efflux pump encoded within the genome of *V. cholerae* is VceB, which is comprised of 14 predicted α-helical transmembrane segments and identified as also having homologous counterparts within the genome of *E. coli* as the operon *emrAB* [179,180]. In *V. cholerae*, the genes encoding an upstream autoregulator (*vceR*), the cytoplasmic membrane pump (*vceB*), an outer-membrane channel (*vceC*), and a membrane fusion protein (*vceA*) are encoded in the *vceCAB* operon of the genome *V. cholerae*. The susceptibilities of *V. cholerae* to nalidixic acid, deoxycholate, phenylmercuric acetate, and carbonyl m-chlorophenylhydrazone increase with the deletion of the gene encoding VceB, suggesting that VceB is capable of transporting these substrates out of the bacterial cell, thereby increasing resistance towards these antimicrobial substrates [180].

##### VcrM Multidrug Efflux Pump

VcrM from *V. cholerae* non-O1 is a multidrug efflux pump with a Na^+^ coupled drug antiporter activity, belongs to the MATE superfamily, and confers resistance to 4',6-diamidino-2-phenylindole, acriflavine, rhodamine 6G, ethidium bromide, and TPPCl [181]. In order to reduce conditions that foster dissemination of these resistance determinants through human populations, it is important to study the MATE drug resistance mechanisms as targets for modulation [182].

#### 2.5.5. Efflux Pumps from *Salmonella enterica*

**Figure 3:**
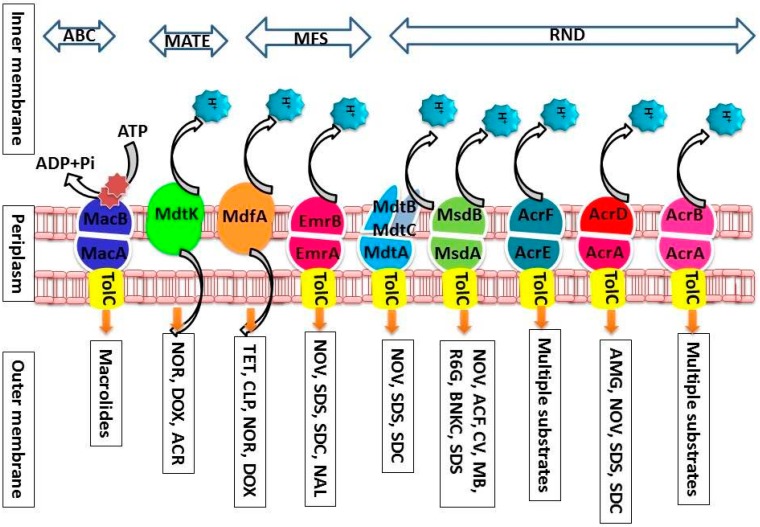
Efflux pumps of *Salmonella* from four different transporter families. The transport mechanisms, location, families and substrates are shown. Abbreviations used in the figure indicate the following: aminoglycosides (AMG), novobiocin (NVB), sodium dodecyl sulfate (SDS), sodium deoxycholate (SDC), acriflavine (ACF), crystal violet (CV), methylene blue (MB), rhodamine 6G (R6G), benzalkonium chloride (BNKC), nalidixic acid (NAL), tetracycline (TET), chloramphenicol (CLP), norfloxacin (NOR), doxorubicin (DOX), and macrolides (MAC).

Multidrug efflux pumps from *Salmonella* (Figure 3) are an obstacle to the treatment of salmonellosis as these transporters extrude structurally different substrates out of the bacterial cell conferring an effective defense against antimicrobials and toxins [13]. Effective modulation and inhibition of antimicrobial drug efflux in *S.* Typhimurium requires knowledge of the molecular mechanism and function of their drug efflux systems and offers an exciting developmental area for drug discovery [12,182]. Experimental evidence has demonstrated increased susceptibilities of *Salmonella* strains to several antibiotics upon deletion of *tolC*, *acrAB acrD*, *acrEF*, *mdtABC*, *mdsABC*, *emrAB*, *mdfA*, *mdtK*, or *macAB* genes [183].

*S. enterica* serovar Typhimurium has nine functional drug efflux pump systems from four major families including, the major facilitator (MFS) superfamily (EmrAB and MdfA); the RND family (AcrAB, AcrD, AcrEF, MdtABC and MdsAB); the multidrug and toxic compound extrusion (MATE) family (MdtK); and the ATP-binding cassette (ABC) family (MacAB) (Table 1) (Figure 2) [184]. TolC is a major outer membrane channel with a major role in the extrusion of antibiotics out of the bacterial cell [184]. Studies measuring the expression of these efflux pumps, and others discussed below, has shown that the known *Salmonella* pump systems confer resistance to erythromycin, novobiocin, chloramphenicol, nalidixic acid, norfloxacin, doxorubicin, acriflavine, crystal violet, ethidium bromide, methylene blue, rhodamine 6G, TPP, benzalkonium chloride, sodium dodecyl sulfate, and sodium deoxycholate [185]. In contrast to these known drug efflux systems in which their physiological properties have been studied in relative detail, *Salmonella* bacteria, nonetheless, harbor many other MFS putative solute efflux pumps for which no physiological data are presently available (Table 1). Thus, much work remains to be conducted in order to eventually understand the functional roles in antimicrobial resistance for these putative efflux pumps belonging to the MFS.

**Table 1:** **Major facilitator superfamily (MFS) efflux pumps of *Salmonella.*** The table shows all the accession numbers, name, description, and database entries of studied and putative MFS efflux pumps found in protein sequence databases. “UP” denotes the UniProt database entry, and “NB” denotes the NCBI entry.

Accession	Pumps	Description	Database
tr|Q8Z810	YCAD	Uncharacterized MFS-type transporter YcaD	UP
tr|P58530	SOTB	Probable sugar efflux transporter	UP
tr|Q8XFG0	MDTM	Multidrug resistance protein MdtM	UP
tr|P37594	SMVA	Methyl viologen resistance protein SmvA	UP
tr|Q8Z257	YHHS	UPF0226 protein YhhS	UP
tr|Q8Z4Z9	YFCJ	UPF0226 protein YfcJ	UP
tr|Q8XGS2	NEPI	Purine ribonucleoside efflux pump NepI	UP
tr|Q8Z7L0	MDTH	Multidrug resistance protein MdtH	UP
tr|P27669	UHPC	Regulatory protein UhpC	UP
tr|P33027	SETB	Sugar efflux transporter B	UP
tr|Q8Z6Q5	DTPA	Dipeptide and tripeptide permease A	UP
tr|P37593	NARU	Nitrate/nitrite transporter NarU	UP
tr|P0A2G3	CITA	Citrate-proton symporter	UP
tr|P40862	PROP	Proline/betaine transporter	UP
tr|C0Q135	Y3650	UPF0226 membrane protein SPC_3650	UP
tr|P33733	TCR4	Tetracycline resistance protein, class D	UP
tr|C0PZN3	NANT	Putative sialic acid transporter	UP
tr|B5R3Y2	Y3404	UPF0226 membrane protein SEN3404	UP
tr|Q8ZLE4	YHHS	UPF0226 protein YhhS	UP
tr|B4SZP4	Y2561	UPF0226 membrane protein SNSL254_A2561	UP
tr|Q5PLW2	TSGA	Protein TsgA	UP
tr|B4T502	LPLT	Lysophospholipid transporter LplT	UP
tr|Q7CQY0	DTPD	Dipeptide permease D	UP
tr|Q8ZLD6	DTPB	Dipeptide and tripeptide permease B	UP
tr|Q57LY2	Y2374	UPF0226 membrane protein SCH_2374	UP
tr|Q57RY1	ENTS	Enterobactin exporter EntS	UP
tr|Q9L7R5	YIHP	Putative 2,3-dihydroxypropane-1-sulfonate exporter	UP
tr|Q9L7R4	YIHO	Putative sulfoquinovose importer	UP
tr|A9N7L0	MDTD	Putative multidrug resistance protein MdtD	UP
tr|B5F0Y5	MdtM	Multidrug resistance protein MdtM	UP
tr|D0ZS93	Multidrug translocase	Multidrug translocase	UP
tr|G4BZY2	Transporter	Major facilitator family protein	UP
tr|E8ZZM6	MdtM	Multidrug resistance protein MdtM	UP
tr|I0A7E0	MdfA	Multidrug translocase MdfA	UP
tr|S5HWQ9	Transporter	Multidrug transporter	UP
B7ZJI2	TetA	*S*GI encoded Tetracycline efflux pump	UP
Q9K2Y4	TetB	*S*GI encoded Tetracycline efflux pump	UP
B0JYJ4	TetG	*S*GI encoded Tetracycline efflux pumps	UP
gi|575533051	CmR	Chloramphenicol resistance pump Cmr	NB
WP_0029541	KDG permease	2-keto-3-deoxygluconate permease	NB
gb|ESG911|	Putative pump	Inner membrane protein	NB
YP_0021121	SeSA_B0056	Hypothetical protein	NB
YP2091	Macrolide-efflux determinant	Macrolide-efflux determinant	NB
WP_0204371	Putative permease	Putative permease	NB
WP_0232341	MdtH	Multidrug resistance protein	NB
YP_0052381	EmrAB	*emrA/ emrB/ tolC* efflux system	NB

##### MFS Efflux Pumps of *S. enterica*

The MFS is the largest characterized family of transporters, found in all living organisms. MFS transporters function as H^+^/drug antiporters as they utilize proton motive force as their source of energy [186].

##### EmrAB of *S. enterica*

EmrAB is an MFS-efflux member that plays a role in the intrinsic drug resistance of *Salmonella*. EmrAB contains the membrane fusion protein EmrA, that facilitates the transport of substrates across both of the inner and outer membranes in a fashion similar to that of MacAB [186]. EmrAB provides resistance against uncouplers. The *Salmonella* EmrAB system confers resistance against novobiocin and nalidixic acid whereas it does not confer resistance to CCCP (Figure 2) [185]*.* The EmrAB efflux pump is a multidrug efflux pump that can efflux substrates nalidixic acid, novobiocin and sodium deoxycholate from the bacterial cell [187]. Over-expression of the *emrAB* genes conferred resistance against novobiocin, nalidixic acid, rhodamine 6G and sodium deoxycholate (DOC) [185]. EmrAB genes are regulated by the EmrR protein, a transcriptional regulator [188].

##### MdfA of *S. enterica*

MdfA efflux pump belongs to the MFS (Table 1). The *mdfA* gene in *Salmonella* encoding the MdfA efflux pump confers resistance to tetracycline, chloramphenicol, norfloxacin, and doxorubicin. Unlike MdfA from *E. coli*, in *Salmonella* the transporter does not confer resistance against acriflavine, ethidium bromide and tetraphenylphosphonium (TPP) [185]. In *E. coli* host cells MdfA does transport hydrophobic drugs like chloramphenicol, ethidium, thiamphenicol, TPP, tetracycline, puromycin, pyronin, ciprofloxacin, norfloxacin, benzalkonium, erythromycin, neomycin, and isopropyl-β-d-thiogalactopyranoside (IPTG). In expression studies of MdfA the transporter confers alkaline-tolerance upon *Salmonella* by catalyzing an Na^+^(K^+^)/H^+^ exchange process in addition to drug/H^+^ exchange [189].

##### The Efflux Pumps of the *Salmonella* Genomic Island I (*S*GI-1)

The efflux pumps from the major facilitator superfamily (MFS) chloramphenicol/florfenicol (*floR* also known as *cmlA*-like) and tetracycline (*tetG*)-encoding ORFs are encoded within these integron sites of *S*GI-1 [190]. The plasmid localized *qnr* gene encodes a fluoroquinolone resistance conferring efflux pump in *Salmonella* Typhimurium DT104 and U302 strains [191]*.* The SG1 region of *S.* Typhimurium DT104 and U302 host the *bla*_Carb2_*, floR* and *tetA(G)* genes [192]. In *Salmonella*, most tetracycline resistance efflux pumps are encoded by the *tetA* genes whereas chloramphenicol efflux pumps are encoded by *floR* or *cml* [193]. TetA(A), TetA(B) and TetA(G) efflux pumps confer resistance to tetracyclines [194]. The *floR* gene provides chloramphenicol/florfenicol resistance, and *cmlA* provides chloramphenicol resistance [172,195].

##### TetA/B/G of *S*GI-1

Tetracycline efflux proteins belong to the major facilitator superfamily (MFS). TetA efflux proteins are membrane-associated pumps that recognize and export tetracycline from the cell (Table 1). The *tetA*, *tetB* and *tetG* genes producing TetA, TetB and TetG efflux pumps, respectively, in *Salmonella* spp. belong to group 1 of the TetA family [196]. The tetracycline resistance proteins Tet(A), Tet(B), and Tet(G) function as metal-tetracycline / H^+^ antiporters. This is an energy-dependent process that decreases the accumulation of the antibiotic in whole cells. Tet(A), Tet(B), and Tet(G) are integral membrane proteins with twelve potential transmembrane domains. The *tetA* gene producing the TetA efflux pump in *Salmonella* spp. has exhibited increased tolerance to tigecycline [196]. The *tetA* gene can be a supplement to *ramR* in *Salmonella* exhibiting the MDR phenotype [196]. Oxytetracycline resistance was conferred by *tetA*, *tetB* and *tetG* in *Salmonella enterica* Typhimurium isolates from cattle and poultry [197,198]. The expression of these *tetA* genes is controlled by a family of tetracycline transcriptional regulators known as TetR. The TetR family of regulators are involved in the transcriptional control of the TetA multidrug efflux pumps [199].

#### 2.5.6. Efflux Pumps from *Enterobacter* spp.

##### EmmdR Multidrug Efflux Pump

The EmmdR multidrug efflux pump from *E. cloacae* has twelve predicted transmembrane segments and some shared identity with members of the multidrug and toxic compound extrusion (MATE) family of transporters [47]. Physiological studies of the antimicrobial agent efflux activities demonstrated that EmmdR is an H^+^/drug antiporter but not a Na^+^ driven efflux pump, indicating that this pump is responsible for multidrug resistance [47]. EmmdR actively pumps out quinolones from cells of *E. cloacae* and is thus an important multidrug resistance mechanism in these bacteria. Homologs of EmmdR include PmpM of *Pseudomonas aeruginosa* [47,200], NorM of *Vibrio parahaemolyticus* [201], and CdeA of *Clostridium difficile* [202].

##### The AcrAB-TolC Efflux Pump System

The AcrAB-TolC efflux pump is involved in multidrug resistance in *E. cloacae* [203]. The structural components of the AcrAB-TolC efflux pump appear to play a role in antibiotic resistance as well as in environmental adaptation and host virulence in clinical isolates of *E. cloacae* [203]. The genes encoding the regulatory proteins SoxS, RobA, and RamA were cloned and sequenced for the first time in this species, and the involvement of these proteins in conferring antimicrobial resistance through the up-regulation of *acrAB* was demonstrated in *E. cloacae* [204].

##### Outer Membrane Proteins OmpD and OmpF

OmpD and OmpF are outer membrane proteins which are considered to associate with the efflux pumps of the AcrAB system [205]. OmpD and OmpF molecules in an ertapenem-resistant isolate were shown to be less permeable than that of a corresponding susceptible control strain, indicating that possession of these two proteins could potentially lead to higher resistance provided by an associated pump system without necessarily requiring expression [205]. OpmD and OpmF from *E. cloacae* share similar structures and functions with OprM and OprN from *P. aeruginosa* [206].

##### SugE Multidrug Efflux Pump

As described above regarding SugE from *E. coli* [207], SugE from *E. cloacae* is similarly a member of the SMR family of transporters [48]. SugE from *E. cloacae* confers resistance to a variety of structurally distinct antiseptics and actively extrudes ethidium bromide [48]. The SugE pump has been demonstrated to translocate H^+^ across the membrane upon the addition of cetylpyridinium chloride or benzalkonium chloride to host cells harboring the determinant [48]. Thus, SugE is a proton-driven secondary active transporter that is demonstrated to be a multiple drug efflux pump, *i.e.*, an H^+^/drug antiporter [48].

## 3. Evolution of Drug and Multidrug Efflux Pumps

A major discovery in the field of solute transport molecular biology was the finding that, from microbes to humans, transporters with structurally dissimilar substrates, such as sugars, amino acids, Krebs’s cycle intermediates, ions, and antimicrobial agents, nonetheless shared similarities in their amino acid sequences and in their predicted 3-dimensional protein structures within the biological membrane [208]. This finding led to the striking prediction that transporters across all taxa with diverse substrates operated to carry a diverse array of solutes and ions across the biological membrane using a common transport cycle or a translocation mechanism [208]. The major facilitator superfamily (MFS) was discovered by Henderson and colleagues [208,209,210,211] and now consists of thousands of transporters with diverse biological origins, substrates, and modes of energy (active *versus* passive), but with similar predicted or elucidated 3-dimensional global protein structures, and mechanisms of transport [212]. Members of the MFS and other transporters are elegantly organized into an extensive Transporter Classification Database http://www.tcdb.org, and investigators may take advantage of many important tools for bioinformatics and biomedical analyses of solute transporters [213]. The MFS transporters have been further sub-divided into families consisting of closely-related members with similar amino acid sequences and numbers of predicted transmembrane domains, such as 12- or 14-membrane segments [101,214].

One of the MFS families, called DHA1 or DHA12, for drug-proton antiporter, contains closely related proton-driven drug efflux pumps that consist of 12 transmembrane domains [215]. Well studied members of the DHA1 family include multidrug efflux pumps NorA from *S. aureus* [122], Bmr from *B. subtilis* [92], LmrP from *Lactococcus lactis* [216], EmrD from *E. coli* [217], MdfA from *E. coli* [218], VMAT1 from *Homo sapiens* [219] and the tetracycline efflux pump systems of the pBR322 plasmid-encoded TetA(C) [220] and the Tn10-encoded TetA(B) protein [221] from *E. coli*.

Another MFS family called DHA2 or DHA14, for drug-proton antiporter with 14 transmembrane domains represents a family related to DHA1 and closely related to each other [215]. Well studied members of this family include the multidrug efflux pump systems Atr1 of *Saccharomyces cerevisiae* [222], EmrB from *E. coli* [223], QacA from *S. aureus* [97], SmvA from *Salmonella enterica*, serovar Typhimurium [224], VceAB from *V. cholerae* [180], SdrM from *S. aureus* [155], MdeA from *S. aureus* [113], and the single-drug efflux pumps for tetracycline TetA(L) from *B. subtilis* [225] and TetA(K) from *S. aureus* [226]. Interestingly, single-drug efflux pumps of the MFS showed homology to multi-drug efflux pumps, predicting that subtle sequence variations in these pumps confer major differences in function, such as substrate number [208]. Despite this intriguing notion, conversion of a single-drug efflux pump into a multi-drug efflux pump by subtle mutation has not been realized, as far as we are aware.

### 3.1. Conservation of Amino Acid Sequence Motifs

**Figure 4:**
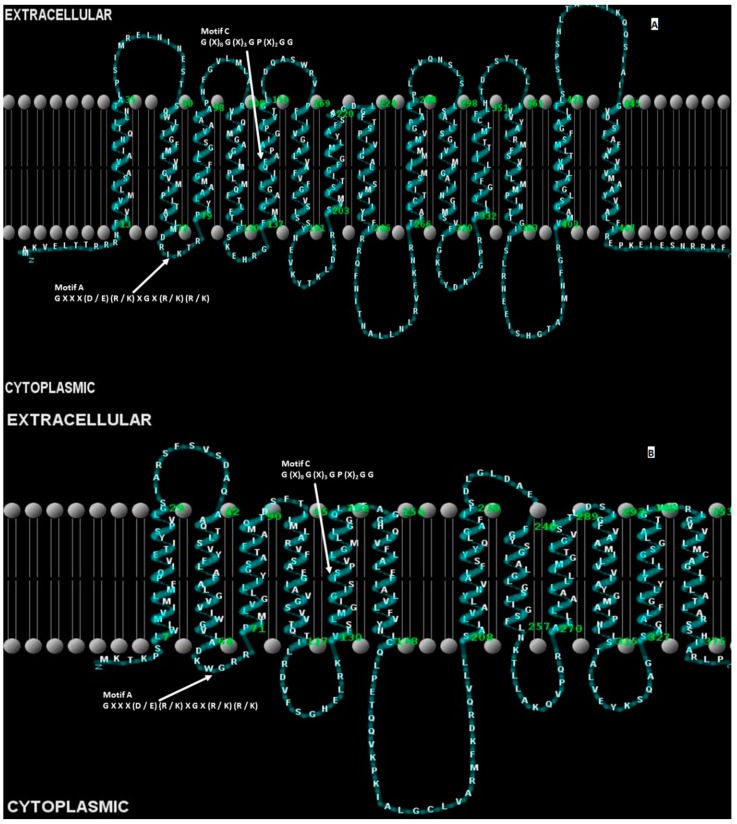
Conserved amino acid sequence motifs A and C of the major facilitator superfamily. The predicted 2D membrane topology structures of the multidrug efflux pumps (**A**) LmrS from *S. aureus* [111] and (**B**) EmrD-3 from *V. cholerae* [177] are shown. The consensus sequence of the highly conserved motif A [97,208], which resides in the loop between predicted transmembrane helices 2 and 3, is “G X X X (D/E) (R/K) X G X (R/K) (R/K).” Likewise, the consensus amino acid sequence of motif C is “G (X)_8_ G (X)_3_ G P (X)_2_ G G” and resides in the fifth predicted membrane spanning domains of most, if not all, transporters of the MFS [81]. Structures were generated using TMHMM and Tmpres2D servers.

An implication of the shared homology between members of the MFS is that conserved amino acid residues within these transporters confer structural or functional attributes in these transport proteins [208]. Extensive primary amino acid sequence comparative studies performed on members of the MFS from a bioinformatics perspective and involving many laboratories have found several categories of highly conserved sequence motifs contained within this superfamily, Figure 4 [95,208,211,212,227].

The first category of conserved amino acid sequence motifs in the MFS involves those motifs found in all or most of the members of a family or sub-family within the MFS [208]. An early example is the motif sequence “G I G L X X P V L P X X L R/K D” conserved within the transmembrane domain 1 of members within the DHA2 family [208]. This region was studied and systematically evaluated using cystiene-scanning mutagenesis in which each residue in the first transmembrane domain of the TetA(B) tetracycline was individually replaced by a cystiene amino acid and assessed for transport activity [228]. In contrast, residues in transmembrane 1 of the lactose permease [229,230] were required for active sugar transport, and residues in transmembrane domain 1 of the raffinose permease of *E. coli* were necessary for substrate selection [231]. Because these types of conserved motifs are independent of direction of transport, mode of energy, or substrate number and type, this suggests that their sequence conservation reflects merely their close evolutionary relatedness between the members of these families [208]. Presently, it is unclear to what extent the residues contained within this motif category are functionally or structurally important as perhaps a motif found not to be relevant in one or more transporters may in fact be critical for activity in another member (or more) of the same family. Further work will be necessary on a case-by-case basis for each solute transporter to definitively demonstrate the functional or structural roles for these types of conserved residues in transporters harboring this category of conserved sequence motifs.

The second category of conserved sequence motifs within the MFS are those that are found within all or most of the members of the very large superfamily [208,211,212,227]. An example of an extensively studied motif in this category, designated motif A, includes the amino acid sequence “G X X X (D/E) (R/K) X G X (R/K) (R/K)” found to reside in the cytoplasmic loop between transmembrane domains 2 and 3 in virtually all members, to some extent, of the MFS (Figure 4) [210,227]. Physiological evidence in support of the functional importance of elements within this conserved motif was first demonstrated in the tetracycline efflux pump, TetA(B), from the Gram-negative bacterium *E. coli* [232,233]. Residues Gly-62, Asp-66 and Arg-70 of the motif were demonstrated to be required for tetracycline transport activity in TetA(B) [234]. Later studies showed that Arg-70 forms a salt-bridge with Asp-120, which resides in the loop between transmembrane domains 3 and 4 of TetA(B) [235,236]. These studies support the notion that the loop between transmembrane segments 2 and 3 interacts with the loop between transmembrane segments 3 and 4 to form a functional gating mechanism during tetracycline/H^+^ antiport [233,237]. Additional evidence of the molecular involvement by residues of the motif was provided by the laboratory of Brooker using the lactose permease, LacY, from *E. coli* [82,238,239,240]. Interestingly, elements of the loop 2–3 motif has, to a certain extent, been duplicated in the loop between transmembrane domains 8 and 9 of a majority of the MFS transporters predicting that a gene duplication and a tandem repeat event occurred phylogenetically of a common ancestor consisting of six transmembrane domains to result in 12 transmembrane domains [210]. Mutational and second-site suppressor analysis of conserved residues residing in the loops formed between helices 2 and 3 and between helices 8 and 9 supported a transport model in which the LacY permease forms a two-fold axis of rotational symmetry in which both halves interact and form the basis of conformational changes at this interface during transport [82], a notion recently supported using a crystal structure of YajR, a drug efflux pump of the MFS, as evidence [241].

The third category of conserved sequence motifs within the MFS are those in which residues are conserved only functionally related sub-groups within the superfamily, such as for example, drug/H^+^ antiporters but not symporters [208]. One example of an extensively studied conserved sequence motif in this category is the antiporter motif, also designated motif C, consisting of residues “G (X)_8_ G (X)_3_ G P (X)_2_ G G” predicted to reside in the fifth transmembrane segment of members in families DHA1 and DHA2, but not in symporters or uniporters, of the MFS (Figure 4) [97,208,242]. The first line of evidence to support the functional role of residues in the antiporter motif was provided for the efflux pump TetA(C) in which Gly-147, adjacent to the conserved proline residue, was analyzed for tetracycline resistance by a systematic replacement with all other possible amino acids where only Gly and Ser were acceptable replacements, residues known to be found in multiple sequence alignments [243]. This work was interpreted such that the molecular structure formed by this motif influences the orientation of the unoccupied substrate binding site (inward-facing for efflux and outward-facing for uptake) and thus confers direction of substrate transport. Further evidence in support of the functional role for residues in motif A was provided in the tetracycline efflux pumps TetA(K) of *S. aureus* [148], TetA(B) of *E. coli* [244], and TetA(L) of *B. subtilis* [245,246], as well as in multidrug efflux pumps Mdt(A) of *Lactococcus lactis* [247], QacA of *S. aureus* [248], CaMdr1p of *Candida albicans* [249], and Mdt(A) of *L. garvieae* [250] and in the neurotransmitter transporter VAChT of *Rattus norvegicus* [251]. Each of these transporters are members of the MFS. More recently, molecular evidence in VMAT2 of *R. norvegicus* indicated that the antiporter motif is part of an overall molecular hinge region consisting of two symmetrical halves, one half composed of interacting helices 2 and 11 and the other half composed of interacting helices 5 and 8, in which the hinge points formed between the two halves mediate conformational changes to provide alternating access to the substrate binding sites on either face of the membrane [81]. This molecular hinge region provides a good target site for modulation by small molecules to disrupt the transport cycle and thus inhibit drug resistance conferred by these homologous efflux pumps [252]. Modification of the parameters for conducting multiple sequence alignments, combined with larger numbers of sequences available for new members of the MFS has found that elements of the antiporter motif are conserved in symporters [81]. In a recently determined crystal structure of the glucose/H^+^ symporter, GLUT-1, of *S. epidermidis*, residues of the fifth helix are believed to play a role in the binding of sugar to the inward-facing conformation and in conformational changes in which helix 5 moves closer to helices 7 and 10 [253]. Thus, elements of the antiporter motif in the fifth transmembrane domain may have widespread, if not universal, importance in the transport mechanism for all members of the MFS.

### 3.2. Conservation of 3-Dimensional Structures

**Figure 5:**
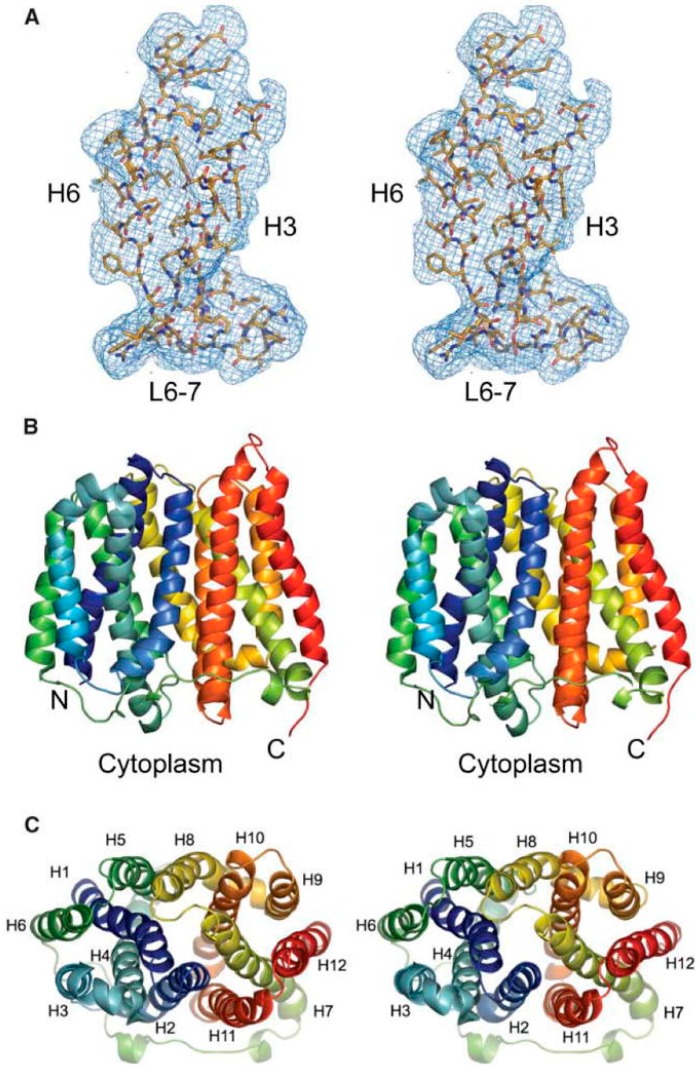
Crystal structure of the EmrD multidrug efflux pump from *Escherichia coli*. The general features of the three-dimensional structure of EmrD include 12 transmembrane α-helices that zig-zag through the inner membrane, a central channel for drug translocation, and a two-fold axis of rotational symmetry by which the antiporters are believed to mediate conformational changes that occur via an alternating access mechanism of the drug binding site. (**A**) An electron density map is shown as a stereo image indicating side-chain densities for α-helices 3 and 6 and the cytoplasmic-facing loop between α-helices 6 and 7; (**B**) The 12 membrane-spanning α-helices are shown in ribbon form as a stereo image; the *N*- and *C*-termini face the cytoplasmic side of the inner-membrane; (**C**) The EmrD structure is shown without loops and from a top perspective looking towards the cytoplasm. The EmrD structure is from Yin, *et al.* [217].

Based on similarities in phylogenetic relationships, primary sequences, and predicted 2-dimensional structures, it has been suggested that although the MFS transporters have differences in the mode of energy and in substrate number and type are, nonetheless, similar in their 3-dimensional structures and, hence, in their mechanism of transport across the membrane [208,211,254]. Of the roughly 10,000 proteins known to be members of the MFS, only about 12, to date, have had their crystal structures solved to high resolution (Figure 5), and these include the following transporters: the oligopeptide-H^+^ symporter, PepT_So_, from *Shewanella oneidensis* [255], the glycerol-3-phosphate transporter, GlpT, from *E. coli* [256], the lactose-H^+^ symporter, LacY, from *E. coli* [257], the nitrate-nitrite antiporter, NarK, from *E. coli* [258], the nitrate-nitrite exchanger, NarU, from *E. coli* [259], the fucose-H^+^ symporter, FucP, from *E. coli* [260], the xylose-H^+^ symporter, XylE, from *E. coli* [261], the multidrug efflux pump, EmrD, from *E. coli* Figure 5 [217], the glucose-H^+^ symporter, GlcP_Se_, from *S. epidermidis* [253], the multidrug transporter, YajR, from *E. coli* [241], the phosphate transporter, PipT, from *Piriformospora indica* [262], and more recently, the glucose-H^+^ transporter, GLUT1, from *H. sapiens* [263].

Comparative analyses of most of the known MFS crystal structures, so far, show that the overall 3-dimensional structures are indeed conserved, such as the asymmetrical halves or bundles, the 12- or 14-transmembrane segments and the *N*- and *C*-termini located in the cytoplasmic side of the membrane [264]. Based on the crystal structures determined thus far all molecular aspects of the known MFS transporters’ structures, however, are not completely conserved, suggesting that the observed differences in molecular structures are responsible for dictating the functional differences between the MFS transporters, such as directions of transport, modes of energy, substrate specificities, substrate numbers, ion specificities, and coupling of ion translocation to substrate transport, in the case of symporters and antiporters [208]. Furthermore, acute knowledge of a transporter’s molecular architecture does not necessarily bestow its molecular mechanism of solute transport across the membrane. For instance, the pathways of the various solutes and ions through the transporters’ channels, cavities or pores, as well as how the transport pathway is established, remain mysterious. Thus, much work remains to be accomplished before the molecular mechanism for transport is clear with respect to the MFS transporters.

## 4. Control of Drug Efflux

### 4.1. Control at the Level of Gene Expression

The emergence of multiple antibiotic resistant pathogenic bacteria has raised concerns about the future of antibiotics in infectious disease control. Among various mechanisms of antibiotic resistance, the increased efflux of antibiotics by the outer membrane proteins has assumed prominence due to their polyspecificity that enables them to expel a large number of unrelated substrates [170]. While many efflux pumps have been discovered in different species of bacteria, their clinical relevance is not fully established for all of them. While some efflux pumps such as AcrAB confer clinical level of resistance, others only marginally increase the resistance of bacteria to target antibiotics [51,265]. The efflux-mediated resistance in many instances is only 2- to 4-fold higher in background strains lacking some major efflux pumps such as AcrAB when the efflux genes cloned in low or medium copy vectors were used. It is not known to what extent these laboratory observations reflect the efflux activities of the strains harboring them *in vivo*, particularly in pathogenic strains. In some pathogens however, efflux pumps have been found to be more active during the antibiotic treatment regime and contribute to elevated resistances to antibiotics [266,267]. This alone, or when combined with other mechanisms of resistance, efflux pumps may confer clinical levels of resistance. For example in *Pseudomonas aeruginosa*, the expression of efflux pumps combined with mutations in topoisomerase genes can increase fluoroquinolone resistance above threshold levels of resistance [268,269]. Similarly, the increased expression of some efflux pumps is associated with decreased expression of porins [268,270]. The activities of efflux pumps lower the intracellular concentrations of antibiotics to sub-lethal levels providing sufficient time for the bacterium to develop other mechanisms of resistance such as mutation in target genes, decreased porin expression, membrane alteration, *etc.* [271,272]. Such events occurring as a result of exposure to one antibiotic are non-specific and can lead to the development of cross resistance to unrelated antibiotics [273,274,275]. Even when no other mechanisms of antibiotic resistance are present in the bacterium, the presence of membrane proteins capable of extruding clinically important antibiotics constitutes potential future threats, since such proteins can evolve to efflux antibiotics to clinically significant levels. Alternatively, the efflux mediated resistance to some antibiotics can result in modification of targets to other clinically important antibiotics due to mutations [269,276,277]. Further, the selective pressure due to exposure to sub-lethal levels of antibiotics can also result in the structural changes in efflux pumps, with the new structural forms being more efficient in efflux of antibiotics [278]. Mutations in efflux pumps or their regulatory genes can enhance the resistance due to expression of efflux pumps and also widen the substrate range for efflux pumps [279,280,281]. The recent surge in the clinical infections by bacteria with efflux-associated MDR phenotypes has changed the earlier view that efflux pumps do not significantly contribute to clinical resistance. The phenomenon of the development of cross resistance to antibacterial compounds unrelated to those bacteria are actually exposed to, has changed the perspective of viewing antimicrobial resistance altogether. Antimicrobial resistances can develop even in the absence of selective pressures by antibiotics. The earlier hypothesis of general target based mechanism of biocides changed with the identification of specific bacterial targets for biocides. For example, Triclosan specifically targets the bacterial fatty acid biosynthetic pathway, enoyl-acyl carrier protein (ACP) reductase (FabI) [282,283,284]. However, triclosan is also a substrate for many efflux pumps of *Pseudomonas aeruginosa*, such as the MexAB-OprM efflux pump [281]. This significant finding suggested that common household disinfectants can confer efflux-mediated cross resistance to clinically important drugs.

The RND superfamily of transporters such as AcrAB-TolC are widely distributed in Gram-negative bacteria and are responsible for resistance to a diverse array of compounds that include disinfectants, dyes, antibiotics, *etc.* Some of the constitutively expressed efflux pumps belonging to the RND group are responsible for the intrinsic resistance to many antibiotics by pathogenic bacteria such as *P. aeruginosa* [269]. Examples of such efflux pumps include the AcrAB-TolC of *E. coli* and MexAB-OprM of *P. aeruginosa* [269,274]. Apart from being responsible for intrinsic resistance to antibiotics, which may be much below the breakpoint MIC levels, these efflux pumps can undergo changes during the course of treatment and bring the MIC levels above breakpoint levels making pathogenic bacteria clinically resistant to a one or more antibiotics.

The increased expression of some efflux pumps is an important factor responsible for the antibiotic resistance in pathogenic bacteria harboring them. This phenomenon has been observed in efflux pumps of the RND type which are able to extrude diverse types of structurally unrelated compounds. The fluoroquinolone resistance phenotype in bacteria is due to mutations in genes encoding DNA gyrase or topoisomerase. However, several efflux pumps are known to confer elevated resistances to fluoroquinolones, as well. Some of the well characterized fluoroquinolone efflux pumps include AcrAB-TolC and QepA of *E. coli* [285,286], MexAB-OprM of *P. aeruginosa* [287], NorA of *S. aureus* [122], and NorM of *V. parahaemolyticus* [288] with homologous efflux pumps being present in diverse Gram-positive and Gram-negative bacteria [289,290]. The expression of efflux pumps is an important factor that contributes to reduced intracellular concentration of fluoroquinolone antibiotics leading to the development of resistance [266,291]. In addition, the poor expression of porin proteins is another important reason for the failure of fluoroquinolones since the antibiotics cannot cross the outer membrane barrier to attain a concentration required for antibacterial activities, reviewed in references [292,293].

Experimental evidence suggests that in clinically important bacteria, expression of efflux pumps leads to resistance against structurally distinct antimicrobials, reviewed in reference [294]. In clinical isolates of *E. coli*, expression of *marA*, a transcriptional activator, correlated with increased fluoroquinolone resistance due to an increased production of AcrAB efflux pump in isolates from urinary tract infection patients [267,295,296]. Yasufuku *et al.* [267] also correlated increased expression of *yhiV* and *mdfA* efflux pumps with the increased MICs of minocycline and sitafloxacin. In *S*. Typhimurium, expression of AcrAB confers multidrug resistance phenotype in clinical isolates [291,297,298]. In AcrAB-TolC overexpressing MDR strain of *Enterobacter cloacae*, inactivation of the *acrA* led to an increase in the susceptibility to a number of antibiotics [203]. The MexAB-OpmrM efflux pump present in *P. aeruginosa* is homologous to AcrAB-TolC which together with MexXY-OprM is constitutively expressed, while the expressions of MexCD-OprJ and MexEF-OprN pumps are rapidly induced by the presence of substrates [299,300]. When homologous efflux pumps are present in a bacterium, the activity of one efflux pump can complement for the absence of a second efflux pump as has been seen with *S*. Typhimurium, in which deletion of AcrB/AcrF resulted in the increased expression of their homologous efflux pump AcrB [301]. In the *Mycobacterium tuberculosis* complex, increased efflux gene expressions is correlated with isoniazid (INH) resistance and efflux pump inhibitors (EPIs) such as verapamil were able to restore the sensitivity to the drug [302].

Of late, evidence for the roles of efflux pumps in conferring MDR behavior in clinical strains are accumulating, strengthening the earlier hypothesis that efflux pumps could confer clinical levels of drug resistance. In many MDR bacteria in which no known mechanisms of antibiotic resistance could be detected, efflux pumps were subsequently found to be responsible for resistance [302]. NorB expression has been reported to contribute to higher ciprofloxacin resistance among blood stream isolates of *S. aureus* [115,303]. Llanes *et al.* [304] reported simultaneous expression of MexAB and MexXY efflux pumps in multi-resistant clinical isolates of *P. aeruginosa*. During the process of treatment, efflux pumps adapt to the drugs used in the treatment and start extruding them at a much faster rate [268,276,305]. This behavior of the efflux pump is attributed to its polyspecificity which enables it to efflux structurally related compounds. The effect of expression is shown in the case of AcrAB-TolC efflux pump which confers elevated resistance to fluoroquinolone antibiotics [306,307]. A direct relationship has been reported between the expression of *acrAB* gene and the MDR phenotype of *E. coli* [266]. The mutations causing increased efflux activity may occur within the structural genes of efflux pumps, promoters or in their regulatory genes. For example, a variety of single mutations in the two-component regulatory system AdeRS can result in the expression of Ade efflux system of *A. baumannii* [308,309]. In *A. baumannii*, expression of AdeABC, AdeFGH, AdeIJK efflux pumps have been reported to be responsible for MDR phenotype of clinical isolates [309,310,311]. Exposure to sub-inhibitory levels of ciprofloxacin, tigecycline and some macrolides lead to the expression of this efflux pump in clinical strains [309,312,313]. Different events such as point mutations in a two-component system *adeRS* that regulates *adeAB* expression or insertion of an IS element upstream of the operon lead to expression of *adeAB*, while mutations in a regulator gene *adeL* is responsible for *adeFGH* expression in clinical isolates [312,314]. AdeFGH, which is responsible for efflux of fluoroquinolone and tigecycline, is clinically important due to its presence in majority (>90%) of *A. baumannii* isolates [309]. Similarly in *Campylobacter jejuni*, expression of CmeB efflux pump enhances resistance to multiple drugs [315,316,317].

### 4.2. Effect on Virulence

In *P. aeruginosa*, expression of either MexCD-OprJ or MexEF-OprN resulted in the reduced production of a T3S and consequently, the impairment of virulence of the bacterium through an unknown mechanism [315]. However, the expression of efflux pumps can be disadvantageous to the host bacterium too and may reduce the bacterium’s fitness and survival. One recent study reports the reduced virulence of *L. monocytogenes* due to the increased expression of MdrT efflux pump as a consequence of a loss-of-function mutation in the transcriptional regulator gene *brtA* that derepresses MdrT [318]. In fact, MdrT and MdrM efflux pumps of *L. monocytogenes* are involved in secreting small secondary messenger molecules cylic-di-AMP (c-di-AMP) which stimulate the secretion of type I interferons which in turn promote *L. monocytogenes* virulence [319,320]. However, an MdrT overproducing strain of *L. monocytogenes* was significantly attenuated in its virulence in a mouse model, the reasons for which were not well known until recently [318]. This finding supports the hypothesis that efflux pumps are not only important for the physiology of the bacterium, but also have a role in the virulence of some pathogenic bacteria. However, such observations must be interpreted with caution. Though in some bacteria efflux pumps contribute to virulence, this may also be an inadvertent consequence of their normal physiological activities. The other proposed activities of efflux pumps that contribute to virulence include secretion of quorum sensing molecules, host adhesion, and survival in the intracellular environment.

In several pathogenic bacteria such as *Salmonella enterica*, *Pseudomonas aeruginosa*, *Klebsiella pneumoniae*, *N. gonorrhoeae* and others, the inactivation of *acrAB*-*tolC* results in reduced virulence due to impaired survivability in host and the environment [185,278,321,322,323,324]. AcrA knockout mutants of *E. cloacae* exhibited reduced virulence and competitiveness in mouse model suggesting that the *acrA* is required for the full virulence and biological fitness of pathogenic *E. cloacae* [203]. In the case of *S. enterica*, Webber *et al.* [325] demonstrated that all components of *acrAB*-*tolC* are required for efficient adhesion to and invasion of epithelial cells and macrophages by *Salmonella* *in vitro*. Several genes essential for intracellular invasion and survival were down regulated in mutant strains lacking *acrAB*-*tolC* [325]. These results have established a direct link between efflux pumps and virulence of pathogenic bacteria. Obviously, pathogenic bacteria overexpressing efflux pumps have a physiological advantage, being able to survive better in adverse conditions, resist antimicrobials and infect the host.

Biofilms formed by many bacteria are resistant to antibiotics and disinfectants and such bacteria exhibit extraordinary fitness and persistence in the environment in which they form the biofilms. Pathogenic bacteria involved in chronic and persistent infections such as cystic fibrosis-associated infections, implant- and catheter-associated infections, chronic otitis medium, and prostatitis, are prolific biofilm formers [326]. The role of efflux pumps in biofilm formation, persistence, resistance to antibiotics and biocides was largely hypothetical, until some recent studies have discovered that expression of efflux pumps contribute to antibiotic resistance by biofilms [327,328,329]. The up-regulation of several *E. coli* efflux pump during biofilm mode of growth has been reported [329]. The higher expression of efflux pumps offers physiological advantage in the form of expelling toxic wastes from the densely populated biofilms, and in the process efflux antimicrobial compounds, which is manifested as enhanced antimicrobial resistances of biofilms. Inactivation of any of the efflux pumps of *S*. Typhimurium by mutation severely affected its ability to form biofilms [330].

### 4.3. Control at the Level of Efflux Pump Activity

Efflux pumps inhibitors (EPIs) are the compounds that interfere with the activities of efflux pumps and therefore are potentially useful in augmenting the actions of antibiotics by allowing them to reach threshold concentrations required for their bacteriostatic or bactericidal activities [331,332,333]. Hypothetically, it is possible to restore the antimicrobial activities of antibiotics which are substrates of efflux pumps, using EPIs as adjuvants with antibiotics, and this idea has now formed into a novel treatment approach termed “combination therapy”. Inhibition of efflux pumps has several advantages as it can lower the intrinsic resistance, increase the efficacy of antimicrobials by reversing the acquired resistance and decrease the frequency of emergence of antimicrobial resistance among bacteria due to mutations and associated structural changes in efflux pumps [334,335].

However, the EPI-efflux pump interactions are very complex, and their pharmacological applications have remained largely hypothetical. Some EPIs are very specific to a particular efflux pump while others can inhibit diverse efflux pumps. For example, phenyl-arginine beta-naphthylamide (PAβN) inhibits RND efflux pumps, while other proton sequestering agents, such as CCCP, inhibit MFS pumps which are potentiated by the proton motive force. A pathogenic bacterium may have multiple efflux pumps belonging to different superfamilies of efflux proteins, which further confounds the choice of EPIs. Nevertheless, since efflux pumps are essential for the normal physiology of the bacterium, any interference in their functions can inhibit the bacterium. Several studies have shown structure-function relationships of efflux pumps and the effect of mutations on the dynamics of substrate transport [182,336,337]. These findings need to be suitably applied to design inhibitors of efflux pumps. A multidisciplinary approach involving structural, chemical and molecular biologists is necessary to discover new therapeutic strategies to combat efflux mediated resistance with potential new targeted drug or inhibitor designing.

In order to identify or design suitable inhibitors of efflux pumps, elucidation of the molecular basis of efflux-mediated antibiotic resistance is critical. Numerous efflux pumps across diverse species of bacteria have been reported but their structures and molecular aspects of efflux functions remain to be understood. Some recent studies have derived the structures of some important efflux pumps by X-ray crystallography and NMR, which will help unraveling the functional aspects of substrate binding and transport, potentiation of efflux pumps, and the role of conserved amino acids among homologous efflux pumps. The crystal structure of AcrB was the first reported crystal structure for an efflux pump [338]. The crystal structures of AcrB and MexB complexed with their substrates have been described, which have helped to elucidate the antibiotic extrusion mechanisms of these efflux pumps [339,340,341].

Recently, several studies have attempted to design efficient inhibitors of efflux pumps. The peptidomimetic compound, PaβN, is a well-studied broad spectrum efflux pump inhibitor which restores the efficacy of fluoroquinolones in Gram-negative bacteria [335]. PAβN inhibits the RND efflux pumps AcrAB of *E. coli* and Mex efflux systems of *P. aeruginosa* [342,343]. Similar observations of efflux pumps being inhibited by EPIs have been reported in *Salmonella* Typhimurium in which PAβN and CCCP effectively inhibited biofilm formation [344]. In *A. baumannii*, PAβN inhibits the efflux of trimethoprim, chloramphenicol and clindamycin by inhibiting the AdeFGH efflux pump [168]. Hannula and Hanninen [345] reported that PAβN could effectively increase the antibiotic susceptibilities of *Campylobacter jejuni* and *E. coli*. Another EPI, 1-(1-naphthylmethyl)-piperazine (NMP), is reported to be an effective inhibitor of efflux pumps in several members of the Enterobacteriaceae, *A. baumannii*, *P. aeruginosa etc.* [311,346,347]. Biofilm formation by *P. putida*, *Klebsiella* spp. and *S. aureus* was substantially reduced in the presence of EPIs such as PAβN and NMP [329].

Since fluoroquinolone antibiotics are preferred substrates of many clinically important efflux pumps, compounds with structural similarities to quinolones can be effective EPIs. Quinolone derivatives such the alkylaminoquinolines can not only restore the bacterial susceptibilities to fluoroquinolones, but also to the structurally unrelated antibiotics [348]. In an MDR strain of *E. aerogenes*, 7-nitro-8-methyl-4-(2-(piperidino) ethyl) aminoquinoline, inhibits the AcrAB–TolC pump and restores its susceptibility to norfloxacin, tetracycline and chloramphenicol [348]. Another quinolone derivative, 2,8-dimethyl-4-(2-pyrrolidinoethyl)-oxyquinoline, has been reported to significantly enhance the susceptibilities of *E. aerogenes* and *K. pneumoniae* to unrelated antibiotics [349].

Inhibitors of the proton motive force such as CCCP are good inhibitors of drug/H^+^ antiporters. However, CCCP has deleterious effects on the bacterial cell membrane since it de-energizes the cell surface by proton sequestration activity [350]. Other proton uncouplers such as omeprazole, rabeprazole, pantoprazole, lansoprazole, esomeprazole are used in the treatment of *Helicobacter pylori* infections along with antibiotics, and a recent study has shown a significant decrease in the MICs of an MDR *H. pylori* isolate using pantoprazole and rabeprazole [351].

Natural compounds of plant origin such as the plant alkaloid reserpine, kaempferol rhamnoside, capsaicin, piperine, methoxylated flavones, isoflavone, porphyrin pheophorbide, silybin, *etc.*, have shown promising results as EPIs [114,127,128,129,352,353,354]. Apart from these, several natural products have been shown to be potent EPIs [331,342,355]. Phytochemicals exhibit least toxicity and hence are more suitable for therapeutic applications. Bioinformatics tools have enabled virtual/*in-silico* high-throughput screening of thousands of phytochemicals for structural similarities with efflux substrates and performance of docking studies with the efflux proteins in order to understand their interactions as shown in a recent study by Aparna *et al.* [356]. With this approach, these authors identified two compounds, lanatoside C and diadzein, as potential inhibitors of the MexAB-OprM and AcrAB-TolC efflux pumps and diospyrone with direct antibacterial activities are shown from *P. aeruginosa* and *E. coli*. In some plant-derived efflux pumps, compounds such as isobavachalcone to be substrates of these pumps, and their activities are enhanced in strains lacking major efflux pumps [357]. This is an important observation and needs due consideration while using phytochemicals as EPIs, since efflux pumps may be the natural defenses of bacteria against antimicrobial compounds of plants. Another interesting study reports that in animals with plant-based diets, EPIs were naturally present; their concentrations often exceeded the levels required for inhibitory activities, suggesting that EPIs might exert selective pressure on the efflux pumps and modulate their transport activities [358].

The presence of multiple efflux pumps that are not uniformly susceptible to inhibition by EPIs has complicated the use of EPIs. As of now, EPIs are not allowed in antimicrobial therapy, and their efficacies as adjuvants with antibiotics need to be studied in greater detail and with a note of caution. Many EPIs mimic the structures of the efflux pump substrates and have identical binding sites. The effects of using efflux pump inhibitors (EPI) over a long period time upon the structure and functions of efflux pumps remain to be understood. The pharmacokinetics of EPIs when used along with chemotherapeutic antibiotics, their toxicities, interactions with non-target efflux pumps in humans and animals, and their mechanisms of actions resulting in efflux pump inhibition need to be extensively studied. With no new antibiotics available in the near future to control the increasing surge of multidrug resistant pathogenic foodborne bacteria, EPIs are increasingly being viewed as potential tools to restore the efficacy of antimicrobial therapy.

## 5. Future Directions

Bacteria from the Gram-negative Enterobacteriaceae and Vibrionaceae families and the Gram-positive *S. aureus* bacterium are formidable foodborne pathogens, and chemotherapy for infections caused by these microorganisms are often indicated. Unfortunately, many of these bacteria are resistant to single or multiple clinical drugs, thus compromising infectious disease treatment. In order to eventually restore the clinical efficacy of chemotherapeutic agents that have been compromised by bacterial resistance mechanisms in order to effectively treat foodborne infectious diseases, it may become necessary to adopt several strategies. Widespread and early education about personal hygiene practices, appropriate hand washing, food preparation and sanitation are critical. Improving the appropriate, or judicious, use of antimicrobials in human and non-human clinical medicine, and agriculture [359,360]. Such antimicrobial stewardship and prudent use programs would definitively help reduce the prevalence of clinical and environmental resistances [361,362,363,364,365,366,367,368]. Recently, a reduction in the clinical usage of prescribed antibacterial agents was followed by a concomitant “disarming” of a multidrug resistant *Streptococcus pneumoniae* “superbug” [369]. Development of novel antimicrobial agents with novel modes of action against bacteria are necessary, as bacteria have developed resistance mechanisms to all known classes of antimicrobial agents [52]. Antibacterial agents with novel cellular targets are needed, such as those that target pathogenic bacterial machinery for homeostasis/housekeeping, metabolism, cell division, energy generation, virulence, gene expression, and drug resistance. Towards this, comparative genomic analysis between pathogens and their non-pathogenic counterparts is a promising avenue for the identification of novel virulence mechanisms in order to realize development of chemotherapeutic agents that target these yet undiscovered mechanisms [178,370]. Progress has been made with respect to modulators of bacterial drug resistance [182,333]. Recently, phage therapy has risen to the forefront of clinical therapy for foodborne pathogens [371,372]. Modulators of bacterial drug resistance may circumvent these mechanisms and restore their clinical efficacy [182,373,374]. Since multidrug efflux pumps of the MFS constitute major resistance mechanisms of foodborne bacteria, these transport systems would make good targets for modulation.

Bacterial resistance due to MFS drug and multidrug efflux pumps systems confound effectiveness of clinically relevant antimicrobial agents. Despite the accumulated wealth of knowledge regarding the protein biochemistry, molecular biology, physiology and molecular structures of the transporters that constitute the MFS, there remain several aspects that require resolution and further work before a clear picture can be gathered with respect to the mechanism of action of these solute transport proteins. A clear understanding of the structure-function relationships of the MFS drug and multidrug efflux pumps [51,183,375,376] will be necessary to design novel inhibitors [331,377,378] and to understand the mechanisms of drug transport inhibition. Furthermore, it will become necessary to gain a full understanding of the mechanism for energy coupling as well as the determination of the uniport *versus* antiport *versus* symport processes, *i.e.*, direction of solute transport [83,230,379]. The molecular mechanisms of substrate and ion specificities of the MFS efflux pumps are poorly understood and require further study. Likewise, the mechanisms that are responsible for determination of substrate number (single or multiple drugs) are unknown at the molecular physiological levels and must be an active area of research. An important avenue would be to unify the known and future crystal structures of the MFS transporters and to correlate them with the actual drug transport pathways through the membrane, including transport catalysis with conformational changes that occur during transport. Further work will be necessary to fully comprehend the mechanisms of gene expression of the bacterial resistance mechanisms in foodborne pathogens. Lastly, analysis of the evolution of the MFS transporters as they migrate through populations during infectious disease outbreaks by food pathogens will be important.
